# Winter and spring atmospheric rivers in High Mountain Asia: climatology, dynamics, and variability

**DOI:** 10.1007/s00382-021-06008-z

**Published:** 2021-10-24

**Authors:** Deanna Nash, Leila M. V. Carvalho, Charles Jones, Qinghua Ding

**Affiliations:** 1 Department of Geography, University of California, Santa Barbara, CA 93106 USA; 2 Department of Geography and Earth Research Institute, University of California, Santa Barbara, CA 93106 USA

**Keywords:** Atmospheric rivers, High Mountain Asia, Orographic precipitation, ENSO, Arctic oscillation, Siberian high

## Abstract

Atmospheric rivers (ARs) that reach the complex terrain of High Mountain Asia (HMA) cause significant hydrological impacts for millions of people. While ARs are often associated with precipitation extremes and can cause floods and debris flows affecting populated communities, little is known about ARs that reach as far inland as HMA. This paper characterizes AR types and investigates dynamical mechanisms associated with the development of ARs that typically affect HMA. Combined empirical orthogonal function (cEOF) analysis using integrated water vapor transport (IVT) is applied to days where an AR reaches HMA. K-means cluster analysis applied to the first two principal components uncovered three subtypes of AR events with distinct synoptic characteristics during winter and spring months. The first subtype increases precipitation and IVT in Western HMA and is associated with a zonally oriented wave train propagating within the westerly jet waveguide. The second subtype is associated with enhanced southwesterly IVT, anomalous upper-level cyclonic circulation centered on 45$^\circ$ E, and precipitation in Northwestern HMA. The third subtype shows anomalous precipitation in Eastern HMA and southwesterly IVT across the Bay of Bengal. Interannual variations in the frequency of HMA ARs and relationships with various teleconnection patterns show that western HMA AR subtypes are sensitive to well-known remote large-scale climate factors, such as the El Niño Southern Oscillation, Arctic Oscillation, and the Siberian High. These results provide synoptic characterization of the three types of ARs that reach HMA and reveal the previously unexplored significance of their contribution to winter and spring precipitation.

**Supplementary Information:**

The online version contains supplementary material available at 10.1007/s00382-021-06008-z.

## Introduction

Atmospheric Rivers (ARs), a term coined in the early 1990s, describes a phenomenon that explains how baroclinic eddies transport large amounts of water vapor via relatively infrequent, long conduits of strong moisture transport across mid-latitudes and into Polar Regions (Zhu and Newell 1994). ARs obtain their high water vapor content from tropical or extratropical moisture sources, moisture convergence, or local evaporation and are often found in the area ahead of the cold front of extratropical cyclones (Rutz et al. 2014; Dacre et al. 2015). Many studies have shown the global importance of ARs to poleward moisture transport, climate, and water budgets (Zhu and Newell 1998; Guan and Waliser 2017; Paltan et al. 2017; Waliser and Guan 2017; Guan et al. 2018; Nash et al. 2018; Ma et al. 2020). Poleward integrated vapor transport (IVT) from ARs makes up over 90% of the total moisture transport in the mid to high latitudes (Zhu and Newell 1998; Guan and Waliser 2015). Studies have also shown ARs are related to precipitation extremes, flooding, seasonal snowpack, and water availability in the western United States and western Europe (Ralph et al. 2006; Guan et al. 2010; Dettinger 2011; Guan et al. 2013; Lavers and Villarini 2013; Wick et al. 2013). Moreover, ARs modulate extreme precipitation and anomalous snow accumulation in many other regions including Antarctica (Gorodetskaya et al. 2014; Bozkurt et al. 2018; Gorodetskaya et al. 2020), Australia and New Zealand (Kingston et al. 2016; Ye et al. 2020; Prince et al. 2021; Reid et al. 2021), the Arctic Ocean and Greenland (Baggett et al. 2016; Hegyi and Taylor 2018; Mattingly et al. 2018; Neff 2018; Wernli and Papritz 2018; Wille et al. 2019; Mattingly et al. 2020), South America (Viale and Nunez 2011; Viale et al. 2018; Ramos et al. 2019), North Africa (Blamey et al. 2018; Akbary et al. 2019; Dezfuli 2020; Massoud et al. 2020), and East Asia (Naoi et al. 2020; Pan and Lu 2020).

While much is known about ARs that simply cross from oceanic regions to land and immediately result in precipitation, not much is understood about ARs that penetrate farther inland (Rivera et al. 2014; Rutz et al. 2014, 2015), as is the case in Southern Asia. ARs in Southern Asia are unique as they spend most of their life cycle crossing land until they encounter the mountainous region surrounding the Tibetan Plateau referred to as High Mountain Asia (HMA). Yang et al. (2018) identified a series of ARs that formed near the equator over the Bay of Bengal between 1979 and 2016 and determined that many were associated with tropical cyclones, and while infrequent, a large proportion of these ARs led to extreme rainfall events in Northeast India, Bangladesh, and Myanmar. Thapa et al. (2018) detected ARs that crossed a transect in Nepal and found that 70% of these ARs were related to extreme rainfall during non-monsoon periods. Both studies indicated that further work is needed to understand the synoptic conditions of ARs in Southern Asia and their modulation by various modes of large-scale climate variability in both monsoon and non-monsoon seasons.

This study aims to examine ARs that reach HMA and their resulting precipitation, which is important to improve our understanding of water resources in Southern Asia, where recent changes in the regional hydrological cycles have been clearly observed over the past decades but the consensus on the causes of these changes has not been reached. It is known that precipitation and the resulting glacial melt in HMA in the spring and summer months provides water resources for hundreds of millions of people in Southern Asia (Hewitt 2005; Kääb et al. 2012). The Karakoram mountain range, located in the western Himalayas, receives approximately 50% of its annual precipitation during winter and spring months from extratropical cyclones or Winter Westerly Disturbances (WWDs) (Bookhagen and Burbank 2010; Cannon et al. 2015; Norris et al. 2017, 2019; Carvalho et al. 2020). Glaciers in the Karakoram have been stable or advancing in a phenomenon known as the ’Karakoram Anomaly’, attributed to increased wintertime precipitation, decreasing summer temperatures, and increases in extratropical cyclone frequency and intensity (Archer and Fowler 2004; Hewitt 2005; Scherler et al. 2011; Bolch et al. 2012; Gardelle et al. 2012; Kääb et al. 2012; Cannon et al. 2015; Forsythe et al. 2017; Norris et al. 2019). Meanwhile, in Central Himalaya, accelerated melting of alpine glaciers has been attributed to increased temperatures, decreasing precipitation, the weakening of the summer monsoon and decrease in nocturnal rains in the mountains induced by the amplification in the anabatic-katabatic winds (Duan et al. 2006; Krishnan et al. 2013; Zhao et al. 2014; Norris et al. 2020). In addition, over the past decades, HMA has been at risk for rainfall-related hazards such as floods, lightning, and landslides that impact nearby populations, infrastructure, and glaciers (Kirschbaum et al. 2010). The role of ARs in driving these rainfall related phenomenon is currently unaddressed.

The objective of this paper is to characterize the climatology of ARs that bring moisture to HMA and result in distinctive regional patterns of precipitation during the non-monsoon months (December–March). This study also investigates the relationships between WWDs and ARs to better understand their interaction with topography and different large-scale climate drivers. To achieve this, we apply combined Empirical Orthogonal Function (cEOF) analysis to daily meridional and zonal IVT anomalies to analyze the variability in synoptic-scale atmospheric fields associated with ARs landfalling in HMA. We also examine the dynamic relationship between HMA ARs and a variety of well-known large-scale climate modes that are most representative and prevailing in the tropics, extratropics, and polar regions over a broad range of timescales, such as the Arctic Oscillation (AO), El Niño Southern Oscillation (ENSO), Siberian High (SH), and Madden-Julian Oscillation (MJO). The organization of this paper is as follows: Sect. 2 describes the data used for this analysis and Sect. 3 outlines the case selection and the methodology used for the cEOF and k-means cluster analysis associated with the AR cases selected. Section 4 outlines the climatology of HMA ARs and identifies synoptic AR subtypes. Moreover, we investigate the influence of climatic modes on the frequency of each ARs subtype and associated synoptic patterns. Results are summarized in Sect. 5.

## Data

**Fig. 1:**
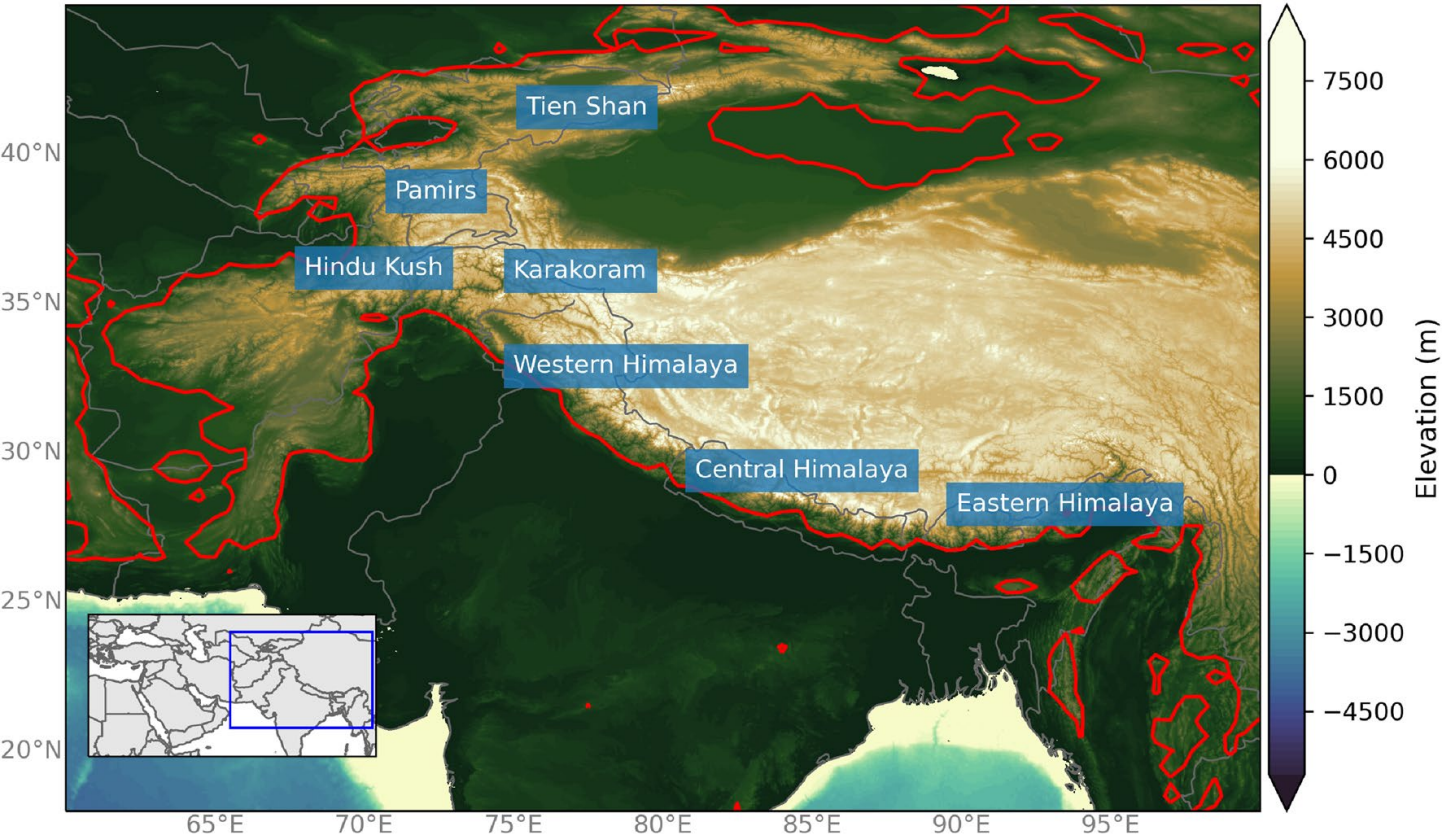
Topographical map of High Mountain Asia (HMA) using the NOAA National Geophysical Data Center’s ETOPO1 1 arc-minute Global Relief Model showing the elevation (contours, m) for the HMA domain. The red line indicates 1000 m elevation using ETOPO1 global relief model upscaled to 0.5$^{\circ }$ horizontal resolution used for identifying HMA ARs. The labels show the relative locations of mountain ranges. The inset map indicates the domain used for the cEOF analysis with the blue bounding box indicated the extent of the topographical map

ARs are identified in this study using a combination of geometry (e.g., length, width), intensity thresholds (e.g., above 85th percentile in HMA), and directional components (e.g., must be poleward), on a 6-hourly global basis between 1979 and 2019 based on the widely used AR detection algorithm introduced in Guan and Waliser (2015), and refined in Guan et al. (2018). The latest version of this algorithm includes tracking capabilities from one 6-hour time step to the next (Guan and Waliser 2019). This AR catalog has been used in many studies and is particularly useful in this study due to the global spatial scale and the application of relative methods for IVT intensity thresholds (Shields et al. 2018; Rutz et al. 2019; Lora et al. 2020). The European Centre for Medium-Range Weather Forecasts (ECMWF) atmospheric reanalyses of the global climate (ERA5) is used here to examine geopotential height, winds, temperature at multiple pressure levels, as well as IVT and precipitation (Hersbach et al. 2020). One main issue in exploring precipitation in HMA is that rain gauges are unevenly distributed and mostly located in lower elevation areas (Andermann et al. 2011; Norris et al. 2017). In addition, complex topography causes challenges in remotely sensed precipitation products, such as systematic underestimation related to the ability of the IR sensors to distinguish between raining and non-raining clouds (Andermann et al. 2011; Palazzi et al. 2013; Behrangi et al. 2016; Maggioni et al. 2016). To account for the uncertainty in these data sets, this research compares precipitation from ERA5 with other precipitation data including the Integrated Multi-satellitE Retrievals for Global Precipitation Measurement (IMERG) V06B and APHRODITE’s (Asian Precipitation - Highly-Resolved Observational Data Integration Towards Evaluation) daily gridded precipitation products (Yatagai et al. 2012; Huffman et al. 2020; Hersbach et al. 2020). In addition, the analysis was repeated using Modern Era Retrospective Reanalysis version 2 (MERRA2) and results were found extremely similar (not shown), indicating the robustness of the results and conclusions (Gelaro et al. 2017). Elevation of the HMA region was determined using the National Oceanic and Atmospheric Administration (NOAA) National Geophysical Data Center’s ETOPO1 1 arc-minute (about 2 km) global relief model (see Fig. 1) (Amante and Eakins 2009). For consideration of AR days, the ETOPO1 data was upscaled to the resolution of the AR Catalog.

We investigated climate modes that are known to influence water vapor content in the troposphere, position and intensity of the subtropical jet, Rossby wave activity, and the storm track in the Northern Hemisphere: AO, ENSO, SH, and MJO. The AO and ENSO indices used for this study were calculated by the National Weather Service Climate Prediction Center (www.cpc.ncep.noaa.gov). The ENSO index is based on the Oceanic Niño Index (3-month running mean of SSTAs in the Niño 3.4 region) (Trenberth 1997). El Niño (La Niña) seasons are identified when the index exceeds +0.5$^{\circ }$ C ($-$ 0.5e$^{\circ }$ C) for at least five consecutive months. The SH index was created by spatially averaging sea-level pressure over the region 80–120$^{\circ }$ E, 40–65$^{\circ }$ N during DJF between 1979 and 2019 and then standardized by subtracting the mean and dividing by the standard deviation of the time series (Panagiotopoulos et al. 2005). For both the AO and SH, conditions are considered positive (negative) when their respective index is 0.5 (− 0.5e) standard deviation above (below) zero. Due to the complexity of the MJO and the wide variety of indices used to identify MJO events, this study uses two different MJO indices. The first is the commonly used Real-time Multivariate MJO (RMM) index which uses cEOF analysis of normalized outgoing longwave radiation, 200 hPa zonal wind and 850 hPa zonal wind meridionally averaged at all longitudes between 15$^{\circ }$ S and 15$^{\circ }$ N (Wheeler and Hendon 2004). MJO composites use phases in which the magnitude of the index is greater than one (Wheeler and Hendon 2004). Daily RMM index values were obtained from the Centre for Australian Weather and Climate Research. The second is the MJO index of Jones (2009), which, in addition to using 20–200 day bandpass-filtered anomalies in the cEOF (same variables as RMM index), defines an MJO event as those that exceed an amplitude of one and have eastward propagation (Jones 2009). These considerations improve the accuracy of the representation of the temporal evolution of MJO events (Jones 2009).

## Methods

### AR events

To focus on ARs that reached HMA, days where an AR reached the 1000 m elevation threshold or higher (between 20$^{\circ }$ N and 40$^{\circ }$ N and 65$^{\circ }$ E and 97$^{\circ }$ E) were considered an AR day in HMA (see Fig. 1). While the number of AR events is sensitive to the choice of elevation threshold, the dynamics explaining these events are not affected. Between 1979 and 2019, we found a total of 1399 AR days in DJF and 1758 days in MAM intercepting the 1000 m elevation threshold. The independence of these events was assessed based on the results of the tracking capabilities from the Guan and Waliser (2019) AR detection algorithm v3. This method uses feature tracking and spatial overlapping from one 6-hour time step to the next to construct the AR tracks (see Guan and Waliser (2019) for more discussion). This resulted in a total of 2278 AR independent events in DJFMAM, which were used to assess the statistical significance tests performed in Sect. 4.3. Approximately half of the events in both DJF and MAM lasted less than 1 day while 10% of the events in both seasons were 5 days or longer. Because of the increased magnitude in water vapor and precipitation and the decreased magnitude in upper-level wind speeds in the spring season compared to the winter season, the seasons were initially separated for the cEOF and k-means cluster analysis. The subtypes of ARs in DJF were extremely similar to the subtypes found in MAM (not shown). Therefore, we discuss the results obtained for the entire DJFMAM season.

### Combined EOF and k-means cluster analysis

A combined EOF analysis (cEOF) was performed to identify the main atmospheric patterns associated with ARs making landfall in HMA. The cEOF analysis was applied to daily meridional and zonal IVT anomalies using ERA5 reanalysis, in a domain extending between 20$^{\circ }$ E to 100$^{\circ }$ E and 10$^{\circ }$ N to 50$^{\circ }$ N for all selected AR days. Since EOF analysis is sensitive to the domain size and choice of variables, several tests were performed to find the optimum domain and the necessary number and type of variables to properly distinguish AR regimes (not shown). We found that despite some variation between number of ARs, variables, and domain choice for the cEOF analysis, results converged indicating the robustness of the analysis. The spatial loadings were calculated (see Appendix A for details) and the distribution of the leading two cEOFs were analyzed (Wilks 2019a). A k-means cluster analysis was further applied to highlight the prominent spatial differences between AR subtypes (see Appendix B for details). Cluster analysis is a form of unsupervised learning that allows for an objective separation of data into groups based on the degree of similarity and differences in the spatial loadings (Wilks 2019b). This methodology can bring out unique grouping of data that might not have been identified, making it a powerful tool for an objective stratification of distinct atmospheric patterns (Cheng and Wallace 1993; Mercer et al. 2012; Peters and Schumacher 2014). The results of the k-means clustering analysis are discussed in Sect. 4.2.

## Results

### Climatology of ARs in High Mountain Asia

The climate of HMA from December to March is mainly influenced by Winter WWDs which are related to extratropical cyclones (Lang and Barros 2004; Bookhagen and Burbank 2010; Cannon et al. 2015). These disturbances are associated with the propagation of troughs and ridges in the upper-level jet stream that can result in precipitation if enough moisture is present when orographically forced (Singh et al. 1995; Filippi et al. 2014; Cannon et al. 2016). There are on average 70 WWDs related to extratropical cyclones per winter and spring season, but only 25% of WWDs result in large-scale precipitation (Hunt et al. 2018). Cannon et al. (2015) suggested that moisture advection was important for extreme precipitation events related to WWDs while Hunt et al. (2018) concluded that rainfall associated with WWDs is related to both orography and the intensity of the WWD. The mechanism behind extreme rainfall related to WWDs still remains elusive and we expect that water vapor transport from less frequent, but impactful ARs is critical to determine seasonal mean precipitation during the winter and spring months in HMA.

**Fig. 2:**
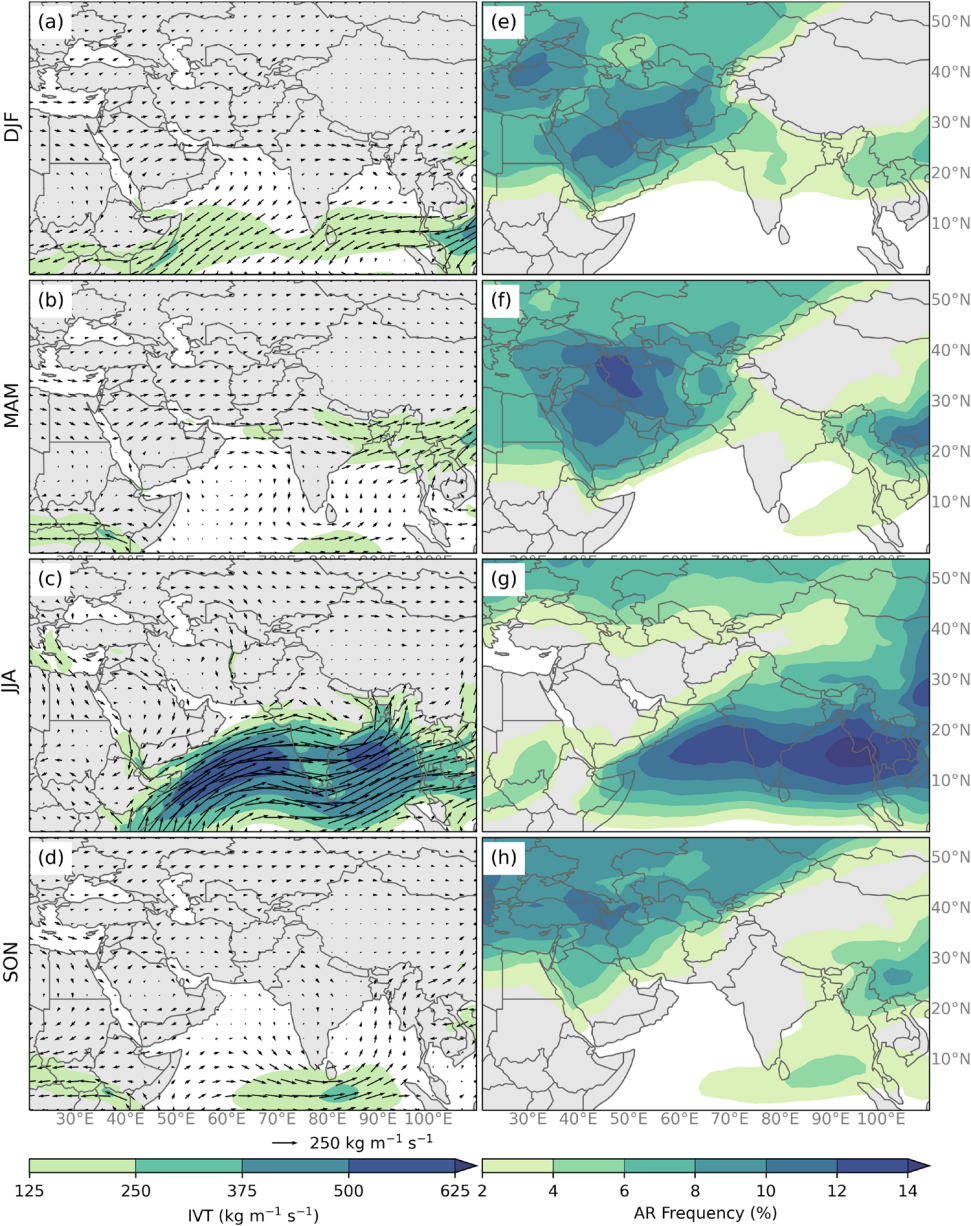
**a**–**d** The average IVT (shaded; kg m$^{-1}$ s$^{-1}$) and IVT direction and magnitude (vectors; kg m$^{-1}$ s$^{-1}$) for each season between 1979–2019 using ERA5 data. (**e–h**) The average AR frequency (shaded; percent of time steps) for each season between 1979–2019 using ERA5 data

A seasonal climatology IVT and AR frequency over Southern Asia using ERA5 reanalysis and the global atmospheric river detection catalog (Guan and Waliser 2015; Guan et al. 2018; Guan and Waliser 2019) for the years between 1979 and 2019 is shown in Fig. 2. During winter (DJF) and spring (MAM) months, the highest frequency of ARs (right column) is observed in subtropical latitudes (between 30–40$^{\circ }$ N), which implies that ARs are associated with extratropical cyclones, otherwise known as WWDs. (Lang and Barros 2004; Bookhagen and Burbank 2010; Cannon et al. 2015; Norris et al. 2019). As expected, during the winter months (DJF) the highest IVTs are observed in tropical latitudes. We also notice an anticyclonic circulation over India, which is typically observed during DJF. Between 30–40$^{\circ }$ N IVT is, on average, less than 200 kg m$^{-1}$ s$^{-1}$ and yet, during DJF there is an AR 8–10% of the time over Southwest Asia. In MAM, IVT begins to increase in subtropical latitudes (to about 150 kg m$^{-1}$ s$^{-1}$) following the transition from anticyclonic to cyclonic circulation over India during the pre-monsoon season, which brings more moisture to subtropical latitudes associated with the intensification of the westerly flow between 20$^{\circ }$ N and 30$^{\circ }$ N. On average, we observe an AR during MAM about 8–12% of the time affecting Southwest Asia and 2–4% of the time in Southeast Asia. To quantify the relationship between extratropical cyclones related to WWD and HMA ARs, we used a catalog developed by Hunt et al. (2018) that identified WWDs over Pakistan and Northern India. This catalog, which extends from 1979 to 2015 identified approximately 2600 WWDs between the months of December to March, averaging about 12 WWDs per month. Therefore, compared to our 2278 AR events, WWDs are just as frequent as HMA ARs (Hunt et al. 2018). When we compared the days with ARs to the days with WWDs based on this catalog, we find that while 73% of ARs occur simultaneously with a given WWD day, only 42% of WWDs are associated with ARs.

Summer (JJA) ARs are typically associated with higher magnitudes of IVT from the Arabian Sea east across Southern India, through the Bay of Bengal and northeast from there. This strong westerly flow and high IVT (exceeding 500 kg m$^{-1}$ s$^{-1}$) indicate a strong association with the summer monsoon flow. Therefore, during JJA, ARs typically affect the Indian Peninsula and adjacent oceans, with a frequency exceeding 10% of the time on average. The higher water vapor transport during the summer season is attributed to increased evaporation over the Indian Ocean and changes in circulation associated with the mature phase of the summer Indian monsoon (Goswami and Mohan 2001; Carvalho et al. 2016; Zhao et al. 2016; Yang et al. 2018). In fall (SON) the highest values of IVT (less than 150 kg m$^{-1}$ s$^{-1}$) are observed in the low latitudes, south of the Indian Peninsula following the progress of the monsoon toward the Southern Hemisphere (e.g. Carvalho et al. 2016). There is an AR between 2–6% of the time during the fall affecting Southwest and Southeast Asia. The frequency of these events also increases over Eastern Europe in the mid-latitudes.

**Fig. 3:**
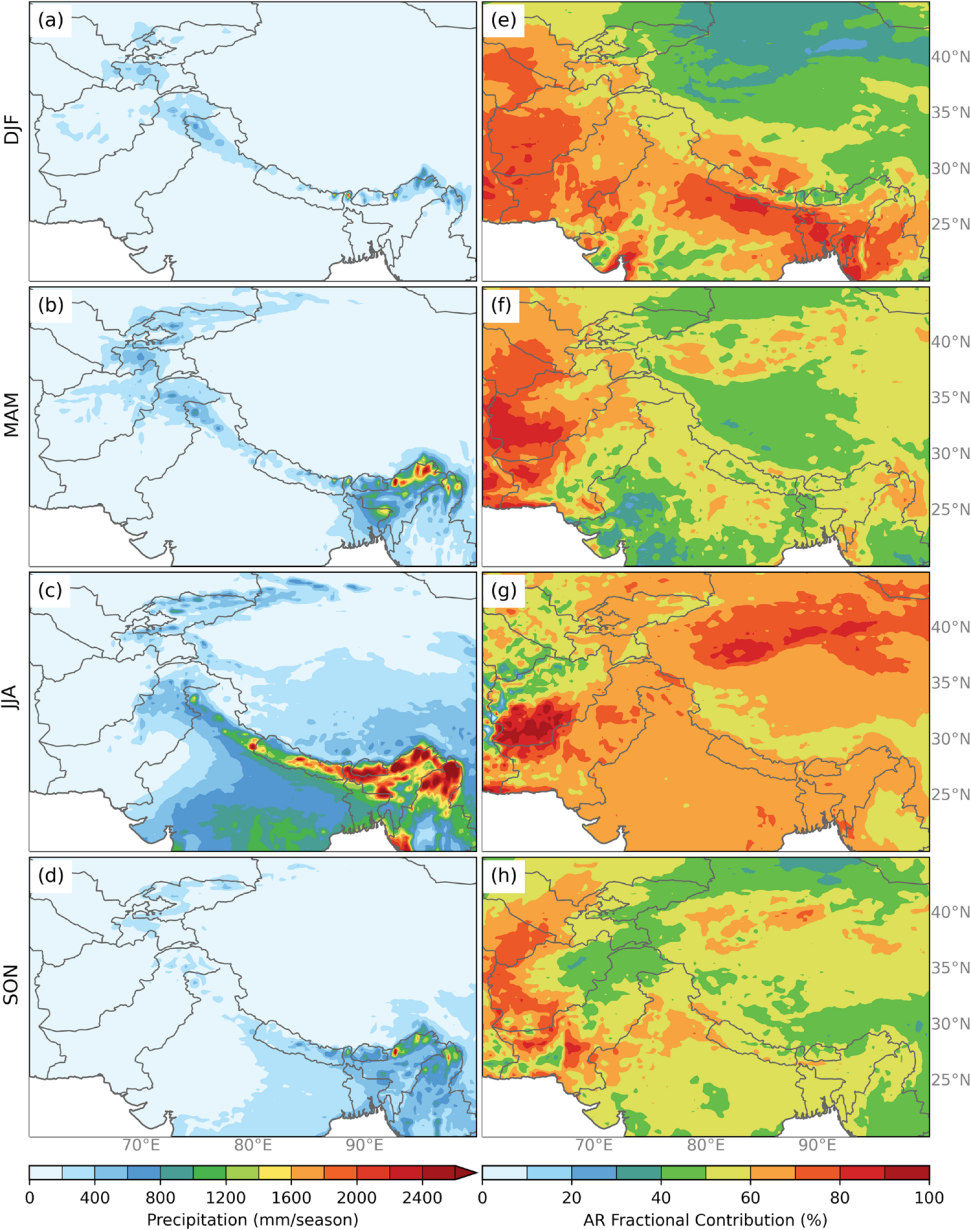
(**a–d**) The average ERA5 precipitation seasonal totals (shaded; mm season$^{-1}$) for DJF, MAM, JJA, and SON. For each grid cell the total seasonal precipitation is calculated per year, then averaged over all years between 1979–2019. **e**–**h** The AR precipitation fraction (shaded, % of total seasonal precipitation) for each season. The total seasonal precipitation that occurs within the AR object is calculated for each season. The AR precipitation fraction is then the total seasonal precipitation that occurs only within HMA ARs divided by the total seasonal precipitation, multiplied by 100 and then averaged over the years between 1979–2019

To determine the relative contribution of HMA ARs to precipitation, Fig. 3 shows the average seasonal total precipitation and the AR precipitation fraction. The AR precipitation fraction is the precipitation (in all grid cells) that occurs on HMA AR days that season compared to the total seasonal precipitation. During the winter (DJF) (Fig. 3a), precipitation is on average between 400-675 mm season$^{-1}$ and occurs primarily in the Pamir Mountains and along the Himalayas at elevations greater than 1000 m. Spring (MAM) (Fig. 3b) precipitation increases particularly in Eastern Himalaya, the Karakoram, the Pamirs, and Tien Shan Mountains (see Fig. 1 for location details). The contribution of precipitation during HMA ARs in DJF is around 60% and about 45% during MAM across HMA. In the area near Mumbai, HMA ARs contribute up to 80% of the total DJF seasonal precipitation (Figs. 3 and S2). However, precipitation from ERA5 indicates high variability (standard deviation) in the high elevation regions and during the spring season (Fig. S3). To check for consistencies, this analysis was repeated with IMERG-PM and APHRODITE (Fig. S1). All three precipitation datasets show high precipitation totals along the Himalayas-particularly in the Karakoram and Eastern Himalaya regions but with varying magnitudes and spatial patterns. ERA5 overestimates precipitation compared to APHRODITE and IMERG-PM and had a higher variance in the eastern Himalayas compared to the other precipitation data sets. Despite these differences, all three precipitation datasets show that HMA AR days contribute roughly 60% of total DJF precipitation and around 40% of total MAM precipitation between 2000 and 2015 along HMA. Interestingly, there is roughly the same contribution of AR events to the total precipitation during the summer monsoon (JJA) and post-monsoon (SON) seasons, indicating the importance of water vapor transport by tropical intraseasonal oscillations (ISOs) in contributing to the total precipitation over the region during the monsoon season.

The climatology in Figs. 2 and 3 show that winter months have much less precipitation in eastern Himalayas and overall a lower magnitude of IVT across Southern Asia compared to spring months. During the winter and spring months in Southern Asia, monsoon circulation weakens and temperatures on the Tibetan Plateau are much colder than temperature at the same altitude over the surrounding oceans, resulting in a typical winter monsoon pattern on India characterized by low-level northeastern winds over the southern Indian peninsula. Meanwhile, the westerly jet is strengthened but retreated equatorward to linger around 30$^{\circ }$ N. This increases the frequency of eastward propagating synoptic circulation systems along the jet such as WWDs from upstream regions toward HMA. (Krishnamurti and Bhalme 1976; Wang 2006; Bookhagen and Burbank 2010). The waviness of the jet combined with topographic influence favors upper-level divergence ahead of the WWD, orographic lifting, and large-scale convection that may result in more interactions between ARs with local precipitation in HMA. In the spring, there is higher atmospheric moisture content due to increased temperatures and saturation vapor pressure in and around HMA at the lower levels, increasing convective instability and the occurrence of precipitation events (Wang 2006; Bookhagen and Burbank 2010). It has been shown that in the spring months preceding the monsoon season, the interaction of extratropical cyclones with the warm moist tropical air mass leads to enhanced moisture advection toward the mountains (Barlow et al. 2005; Cannon et al. 2017). These warmer, moister, and less stable atmospheric conditions combined with orographic forcing can work together to result in more active roles of ARs in triggering intense precipitation over HMA. It seems that more attention should be placed on the impact of ARs, moisture availability and other key atmospheric variables that critically control moisture advection in the seasons.

### Defining patterns of variability of AR events

Combined EOF (cEOF) analysis followed by k-means cluster analyses were applied to identify the main synoptic patterns associated with the occurrence of AR events (see Sect. 3.2 and Appendix A). Daily meridional and zonal IVT anomalies from ERA5 reanalysis were used between 20$^{\circ }$ E to 100$^{\circ }$ E and 10$^{\circ }$ N to 50$^{\circ }$ N for the cEOF. To test the influence of seasonality on the cEOF and k-means cluster analysis we separated DJF and MAM AR days. While magnitudes of the resulting composites vary between the two seasons, the cEOF spatial patterns and the resulting synoptic conditions of the subtypes identified in the separate seasons were very similar. Therefore, results presented included cases in both seasons (hereafter, DJFMAM).

**Fig. 4:**
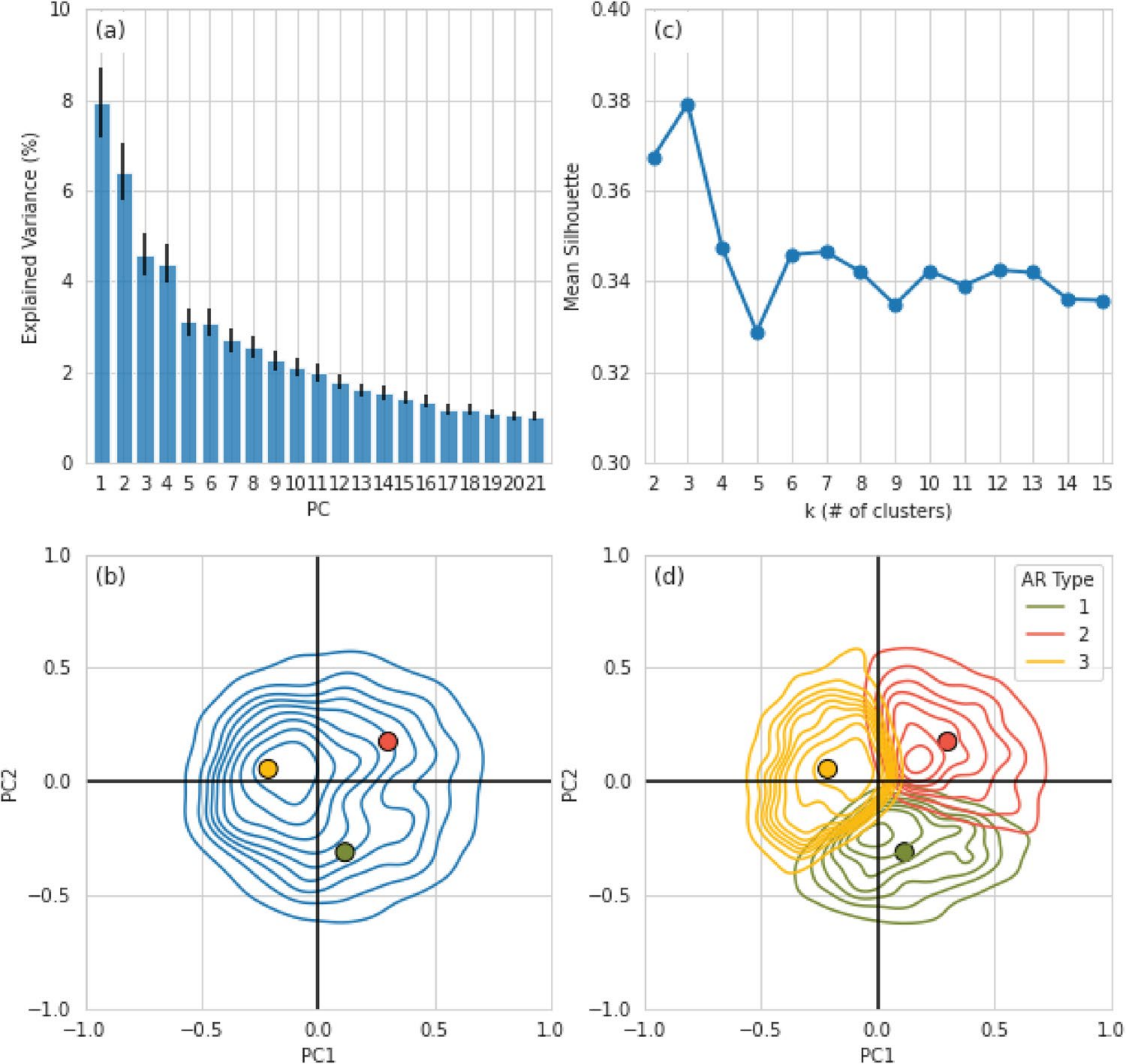
**a** Percent of variance explained by the first 21 DJFMAM PCs and their associated error calculated using the North Test (Wilks 2019a). **b** Estimated kernel density function for PC1 and PC2 loadings for the ERA5 data analyzed for all AR events (contour lines) during DJFMAM. The three points indicate the PC loadings for three specific AR days that were categorized as different AR types during the k-means cluster analysis. **c** The mean silhouette score for clusters k = 2 through k = 15 (blue line). The ideal number of clusters chosen is where the mean silhouette score is the highest (Wilks 2019b). **d** Same as **b** but broken down by AR Types after the k-means cluster analysis. The points in **d** are the same as those in **b**

**Fig. 5:**
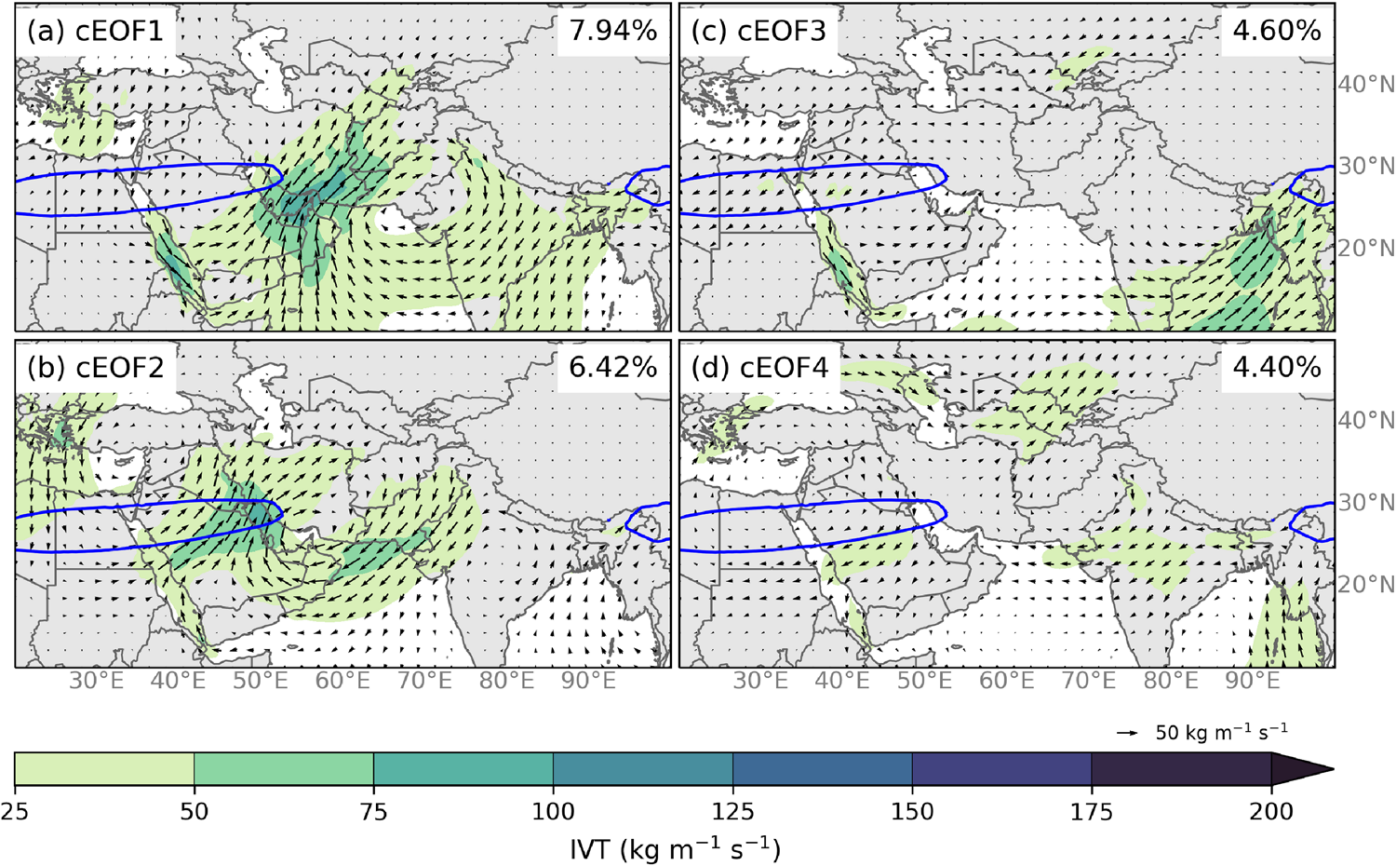
The first four eigenvectors of cEOF analysis for the mean IVT (shaded, kg m$^{-1}$ s$^{-1}$), zonal, and meridional IVT (vectors, kg m$^{-1}$ s$^{-1}$) anomalies during HMA AR days in DJFMAM at gridpoints in southern Asia. The percentages in the top right of each panel show the fraction of the total percent of variance represented by each of the corresponding cEOFs. The blue line indicates the mean position of the core of the 250 hPa subtropical jet ($\ge$ 40 m s$^{-1}$)

Figure 4a shows the fraction of the total percent of variance represented by each of the corresponding PCs. The first two PCs of the correlation matrix for the mean meridional and zonal IVT anomalies during HMA AR days in DJFMAM account for approximately 14% of the normalized DJFMAM IVT variance. According to the North (1984) test, only the first 2 cEOFs were sufficiently separated. PC3 and PC4, while not separated from each other, do appear to represent distinct variance and were included during the testing phase of the k-means clustering analysis to determine if they were necessary to retain. There was little variation in the final results when PC3 or PC4 were included in the k-means clustering analysis. Therefore, only the first two PCs were retained for the final analysis. The spatial loading patterns of the first four cEOFs (see Fig. 5) reflect the underlying circulation patterns in the atmosphere during these HMA AR events. For example, cEOF1 reflects the pattern of a strong anticyclonic anomaly centered around the northeastern Arabian Sea with strong southwesterly flux across Southwest Asia that reaches roughly 40$^{\circ }$ N. While the second cEOF is also associated with above-average southwesterly flux over Southwest Asia the anticyclonic anomaly is located west of the anticyclone in cEOF1, and the IVT doesn’t reach as far poleward as cEOF1. In addition, cEOF2 shows a cyclonic anomaly centered over the eastern Mediterranean Sea that appears to be interacting with the anticyclonic anomaly.

To objectively characterize spatial differences between AR subtypes we applied the k-means cluster analysis to the first two PCs (see Sect. 3.2 and Appendix B). To estimate the optimal number of clusters, we examined the estimated kernel density function for the subspace of the first two PCs (Fig. 4b) (Peters and Schumacher 2014). The kernel density estimate of the first two PC loadings exhibits one distinct maxima for all cases in DJFMAM approximately centered in a region with negative PC2 and biased toward a positive PC1 loading (yellow point, Fig. 4b). There are two additional less distinct maxima (green and red points, Fig. 4b) that are related to positive (red point) and negative (green point) PC2 loading. To ensure the robustness of the choice for the number of clusters, we also calculated the mean silhouette scores, which display how close each point in one cluster is to points in the neighboring clusters (Wilks 2019b). A mean silhouette score of 1.0 would indicate that the sample is far away from other clusters. By reiteratively running the k-means clustering with k values from 1 to 15 and identifying the highest mean silhouette score, 3 was identified as the optimal k for both seasons (see Fig. 4c). Additionally, we performed sensitivity tests for k = 2 and k = 4 by performing numerous composites (not shown) and concluded that k = 3 was the optimal number of clusters for the selected HMA AR cases. Figure 4d shows the kernel density estimate using the identified 3 clusters from the k-means cluster analysis with each subtype having a unique centroid. All subtypes had sufficient sample size to break them into 3 clusters (n = 857, 886, and 1414 for AR Type 1, 2, and 3, respectively).

**Fig. 6:**
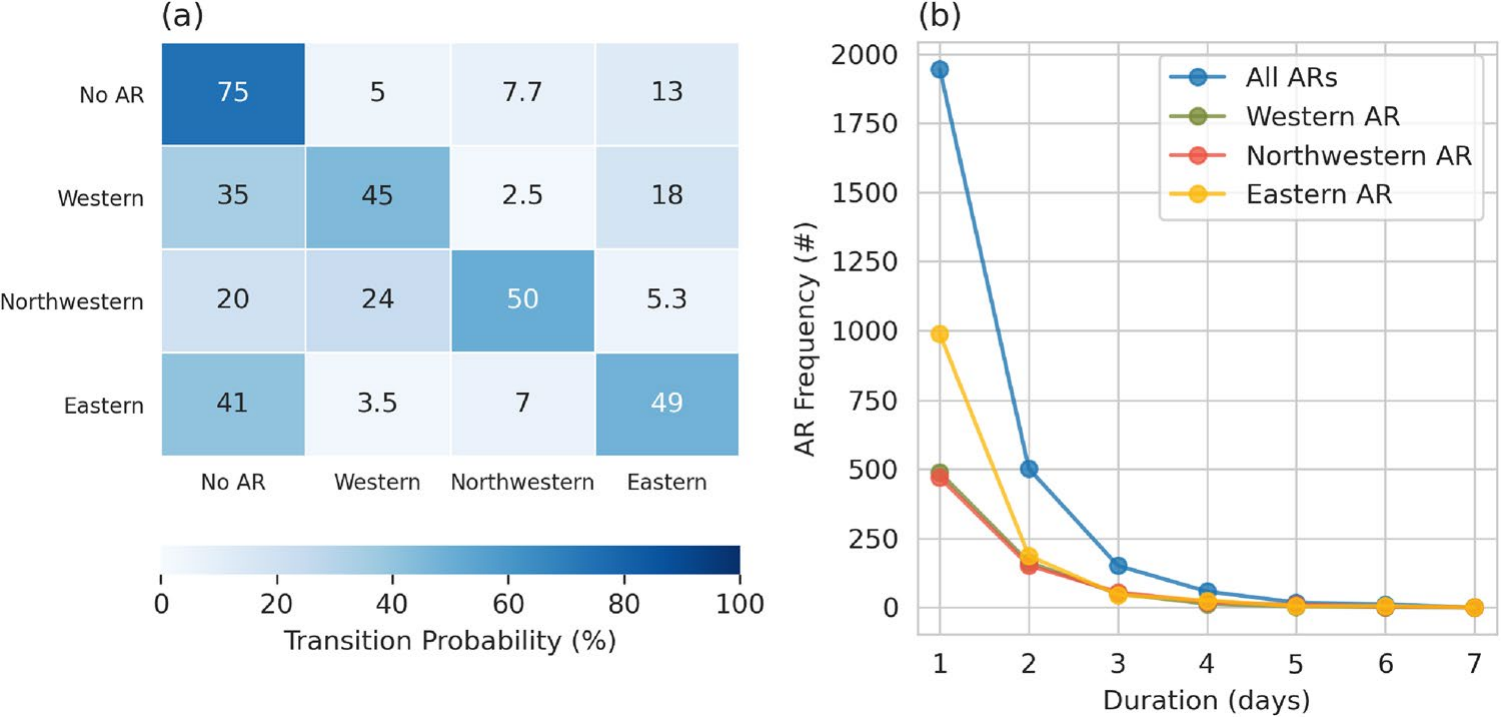
**a** The state transition probability matrix for DJFMAM days that start in state 0 (no AR, first row), state 1 (Western HMA AR, second row), state 2 (Northwestern HMA AR, third row) and state 3 (Eastern HMA AR, fourth row) and ends up in state 0 (no AR, first column), state 1 (Type 1 AR, second column), state 2 (Type 2 AR, third column), or state 3 (fourth column). The transition period is 1 day and the probability is given in percent. **b** The number of ARs and their respective duration (days). The blue line represents all ARs, green is Western HMA ARs (Type 1), red is Northwestern HMA ARs (Type 2), and yellow is Eastern HMA ARs (Type 3)

To evaluate the temporal relationships among AR subtypes for both seasons, transition probabilities were computed to determine the likelihood of each AR Type transitioning to another AR Type or to a non-AR day. Figure 6a shows the probability that a Type 1 AR, Type 2 AR, Type 3 AR, and non-ARs will transition to another type of AR (or non-AR) the next day. In all cases there is a 45% to 50% chance that any AR Type will remain in the same AR type the next day. In some occasions, AR types transition from one type to another. For instance, the probability that Type 2 AR would transition to a Type 1 AR is higher than to Type 3, while the probability that Type 1 AR would transition to a Type 3 AR is higher than to Type 2. Nonetheless, ARs are typically transient (half of the ARs last 1 day or less) and there is between 20% to 40% chance that any AR type will transition to a non-AR the next day. The next section will describe how the results from the combined EOF and k-means cluster analysis were used to create synoptic composites for each of the unique AR subtypes.

### Characterization of synoptic subtypes

**Fig. 7:**
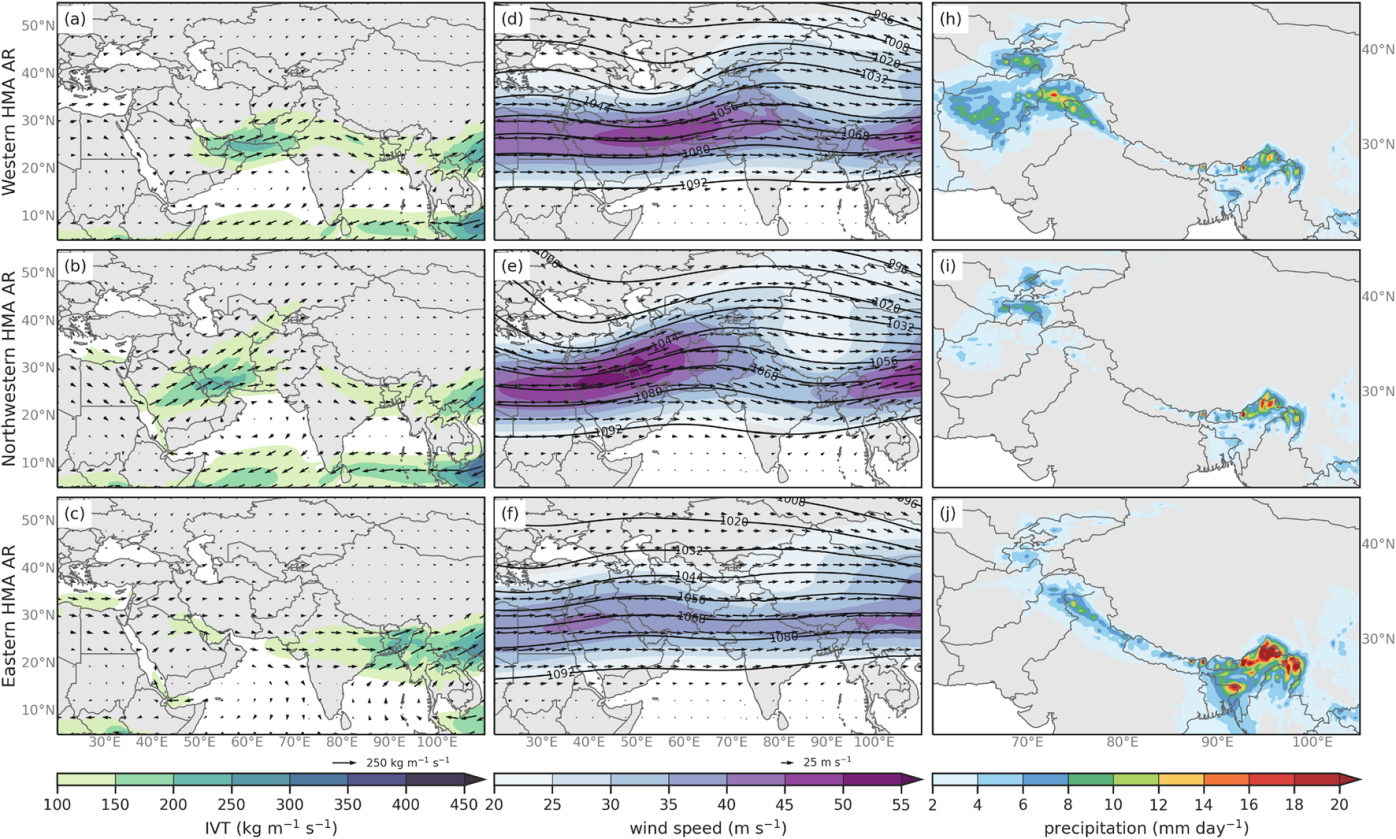
DJFMAM average composites of (left column) IVT (shaded, contours, kg m$^{-1}$ s$^{-1}$), (middle column) 250 hPa wind speeds (shaded and vectors; m s$^{-1}$) and 250 hPa geopotential height (contours; dam), and (right column) precipitation (shaded; mm day$^{-1}$) for Western HMA ARs (Type 1, first row), Northwestern HMA ARs (Type 2, second row), and Eastern HMA ARs (Type 3, third row)

**Fig. 8:**
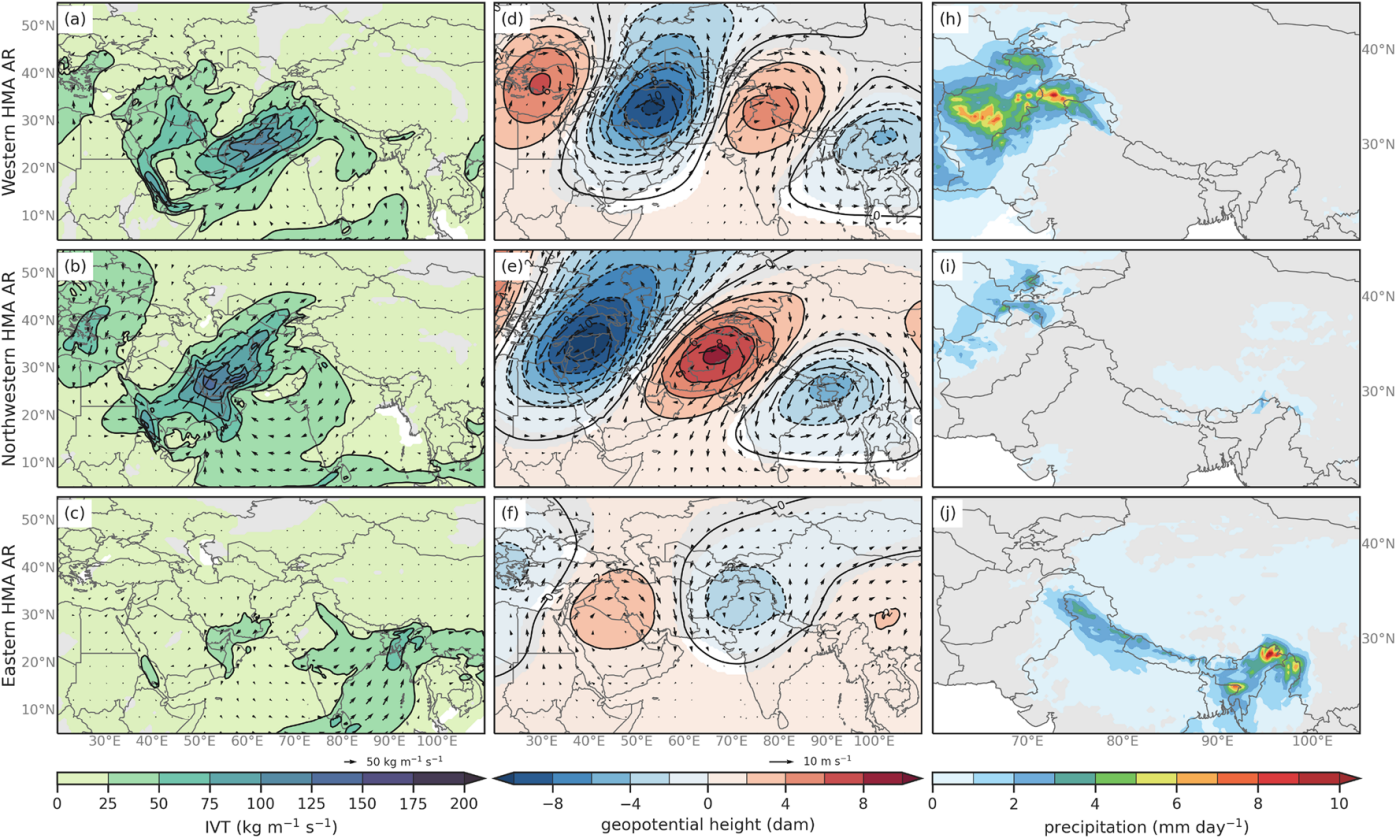
DJFMAM average anomaly composites of (left column) IVT (shaded, contours, kg m$^{-1}$ s$^{-1}$), (middle column) 250 hPa wind speeds (shaded and vectors; m s$^{-1}$) and 250 hPa geopotential height (contours; dam), and (right column) precipitation (shaded; mm day$^{-1}$) for Western HMA ARs (Type 1, first row), Northwestern HMA ARs (Type 2, second row), and Eastern HMA ARs (Type 3, third row). Only values that are considered statistically significant at the 95% confidence interval are shaded

Synoptic conditions associated with AR subtypes were characterized by performing composites of mean (Fig. 7) and anomalies (annual cycle removed) (Fig. 8) for IVT, 250 hPa geopotential heights and winds, and precipitation. The most notable differences between the three subtypes can be identified by the location and strength of the subtropical jet and geopotential anomalies, as well as the intensity and direction of the IVT. Type 1 ARs are associated with anticyclonic flow of above average IVT over southwest Asia and result in precipitation in Hindu Kush and Karakoram mountain regions. Type 2 ARs are characterized by southwesterly IVT over southwest Asia and above average precipitation in the Zagros, Pamir and Tien Shan mountain regions. Type 3 ARs are characterized by southwesterly IVT that is mostly sourced from the Bay of Bengal and Northern India and results in precipitation across HMA, particularly in eastern Himalaya. For simplicity, we will refer to the ARs by the general location of the majority of their resulting anomalous precipitation. For example, Type 1 ARs will be referred to as Western HMA ARs, Type 2 ARs as Northwestern HMA ARs, and Type 3 as Eastern HMA ARs for the remainder of the paper. The seasonal frequencies of these events (see Fig. S4) show that Western and Northwestern HMA ARs are most frequent in March, with all three types occurring up to 250 times between 1979 and 2019. This is important as March is considered a transition month and interannual variability in temperatures (particularly freezing levels) can impact the amount of rain versus the amount of snow. Eastern Himalayan ARs dominate in May, where there has been over 350 ARs between 1979 and 2019.

Western HMA ARs (Type 1, Fig. 7, first row) are identified by IVT with a westerly flow centered around 25$^{\circ }$ N and above-average precipitation in the Karakoram and Hindu Kush mountain regions with precipitation reaching between 16–20 mm day$^{-1}$ in the Western Himalayas and the Karakoram. The subtropical jet for Western HMA ARs is weaker along HMA on average compared to Northwestern HMA ARs. Western HMA ARs are associated with anomalous trough and cyclonic circulation located at about 30$^{\circ }$ N and 60$^{\circ }$ E at 250 hPa and enhanced southwesterly moisture flux across northwestern India (see Fig. 8). Western HMA AR IVT has a more southwesterly orientation for the two days prior to the AR crossing the 1000 m threshold, but then has a more zonal orientation two days after, resulting in above average precipitation in the Karakoram and Western Himalayas (see Figs. S5 and S6). Some ARs that were identified in winter and spring months in Thapa et al. (2018) are considered Western HMA ARs (e.g., 5 Feb 2013, 5 Mar 1980, and 17–19 Feb 2003, among others). Thapa et al. (2018) concluded that these AR events are the main mechanism for bringing moisture into HMA in non-monsoon months and they associate them with most extreme precipitation events in non-monsoon months. The detection algorithm employed in Thapa et al. (2018) focused on ARs that crossed a transect in Nepal, and they concluded that these types of ARs were less likely to occur in MAM compared to DJF due to lack of synoptic forcing. Our results show that Western HMA ARs are more likely to occur in MAM than DJF, though discrepancies between this work and Thapa et al. (2018) are most likely to differing detection algorithms and study region.

Northwestern HMA ARs (Type 2, Fig. 7, second row) are associated with enhanced southwesterly IVT that brings moisture from up near the Red Sea, across southwest Asia and the Persian Gulf and northwest into Hindu Kush, Pamirs, Karakoram, and Tien Shan regions. Northwestern ARs result in up to 8 mm of precipitation per day in the Pamirs, and up to 20 mm day$^{-1}$ in Eastern Himalaya. Anomaly composites show that Northwestern ARs result in above-average precipitation in the Pamirs associated with a trough centered around 40$^{\circ }$ N and 40$^{\circ }$ E and a ridge centered at 30$^{\circ }$ N and 70$^{\circ }$ E that are 100 m below and above average heights respectively (see Fig. 8). The wave train from Central Europe to East Asia also appears to encourage northwesterly moisture flux from the Mediterranean Sea to the Red Sea, where the tilted trough then facilitates southwesterly vapor transport straight to the region surrounding Western Himalayas. In addition, anticyclonic IVT anomalies over the Arabian Sea funnel even more moisture poleward toward HMA. While there were no significant trends for HMA AR frequency, Smith and Bookhagen (2018) shows evidence for increases in snow-water-equivalent storage in the northwestern region of HMA during DJF and in the high-elevation regions of the Pamirs and the Karakoram during MAM. These changes are attributed to increases in precipitation due to higher intensity WWDs in the most recent years and could be related to the ’Karakoram Anomaly’ (Smith and Bookhagen 2018). It is possible that Northwestern HMA ARs could be related to the growing glaciers in the northwestern HMA region. Although we found no significant trend in the frequency of different AR Types, further work is needed to investigate how dynamical and thermodynamical features have changed in recent decades with regard to ARs. Precipitation in Eastern Himalayas during Northwestern HMA ARs could be attributed to the trough over Northeast India, which encourages the transport of moisture towards Eastern Himalayas. Roughly 15% of the time that there is a Northwestern HMA AR, an additional AR in Southern Asia transporting moisture to the Eastern Himalayas was identified. However, this moisture transport and resulting precipitation in Eastern Himalayas during Northwestern HMA ARs is not considered significantly above-average.

Some Northwestern HMA ARs have also been found to extend backwards across northern Africa. Massoud et al. (2020) documented an AR on January 25, 1994 that stretched over 12,000 km from western North Africa to the Karakoram. This was an anomalous case as the majority of the other ARs that impact the Middle East and Northern Africa were found to originate over the North Atlantic and dissipate in Iran where the Zagros Mountains in western Iran reach heights of 1250 m (Massoud et al. 2020). Another example of a Northwestern HMA AR that traversed northern Africa and ultimately ended in western Himalayas was documented in Dezfuli (2020) as well as Massoud et al. (2020). This AR, named “AR Dena”, resulted in widespread flooding, particularly in Iran (Dezfuli 2020). Dezfuli (2020) suggested that the moisture in the AR was enhanced due to warmer than normal marine basins that the AR passed over. While it is curious how Northwestern HMA ARs are able to traverse the complex topography of southwest Asia so far inland, the deep synoptic troughs along southern Iran and Pakistan appear to allow for moisture from marine basins, such as the Persian Gulf and Arabian Sea, to reinforce the aggregation of water vapor that is then transported all the way to the Pamir Mountains via cyclonic circulation as suggested in other regions where ARs persist inland (e.g., Cordeira et al. 2013; Dezfuli 2020). Then, as moisture-filled air in the AR is forced orographically, precipitation most likely develops on the windward side of the mountains.

Eastern HMA ARs (Type 3, Fig. 7, third row) are characterized by IVT from northern India extending eastward resulting in precipitation in the eastern Himalayas. The 250 hPa geopotential heights and winds for these ARs indicate the subtropical jet is located between 20$^{\circ }$ N and 35$^{\circ }$ N with a jet streak occurring across southwest Asia. Eastern HMA ARs coincide with a 250 hPa trough centered around 30$^{\circ }$ N and 75$^{\circ }$ E that is about 20 m below average height and ridge to the west of the trough that is about 20 m above average height (Fig. 8). These ARs are associated with above average southwesterly vapor transport from the Bay of Bengal region up to the Eastern Himalayas which most likely resulted in above average precipitation from terrain effects (see Fig. 8). Some ARs that were identified in winter and spring months in Yang et al. (2018) are considered Eastern HMA ARs in this analysis. Yang et al. (2018) suggests that the majority of these ARs are heavily influenced by tropical activity, where tropical cyclones can enhance longitudinal transport of water vapor in the ARs. Additionally, they concluded that a high proportion of December and January ARs in the Bay of Bengal led to extreme rainfall (Yang et al. 2018).

The synoptic conditions associated with all three HMA ARs have similarities with WWDs. What makes HMA ARs unique from WWDs is the enhanced moisture content within the AR. Cannon et al. (2015) investigated extreme precipitation events in the Karakoram and Central Himalaya and found that, while similar synoptic conditions were characteristic of WWDs for both regions, events that resulted in extreme precipitation in the Karakoram were independent from events that resulted in extreme precipitation in Central Himalaya. The main difference between the two types of events was the position of the trough, location of the moisture source, and precipitation. Our results show similar conditions for Western HMA ARs and Karakoram extreme precipitation, whereas Eastern HMA ARs resemble synoptic conditions that caused extreme precipitation in Central Himalaya in Cannon et al. (2015). Synoptic conditions during Northwestern HMA ARs resemble conditions leading (-2 days) extreme precipitation in the Karakoram region in Cannon et al. (2015), which aligns with the 24% probability that Northwestern HMA ARs transition to Western HMA AR within one-day (see Fig. 6). Further complicating matters, there can be more than one AR reaching HMA at any given time. For example, roughly 15–17% of any AR type occurs simultaneously with an additional AR in the Southern Asia region, indicating the complexity of HMA ARs compared to ARs in other regions of the world.

The median IVT within all three AR Types is roughly 200 kg m$^{-1}$ s$^{-1}$ and 50% of each AR type contains IVT between 100 and 300 kg m$^{-1}$ s$^{-1}$. A little under half of the events for each AR type falls outside the interquartile range, with very few events reaching up to 700 kg m$^{-1}$ s$^{-1}$, indicating the large variability of these ARs (see Fig. S4b). Similarly, precipitation for each AR Type shows large variability, with less than 25% of each type contributing a maximum of 55–115 mm per AR event. In the area they are most likely to see above-average precipitation, the majority of ARs in each subtype results in less than 10 mm per event of precipitation (see Fig. S4c). For example, for Eastern HMA ARs, if you area-average the total precipitation per AR event around Eastern HMA (24–30$^{\circ }$ N, 90–100$^{\circ }$ E), the majority of ARs contribute between 1 and 10 mm event$^{-1}$. This confirms what previous research has said about ARs—impactful ARs are relatively infrequent and just a few AR events per season can lead to significant contributions to water resources where they make landfall.

### Climate modulation

Using the HMA AR climatology presented in this research, we investigate the influence of ENSO, AO, SH, and MJO on the frequency and intensity of HMA ARs during the winter and spring seasons. On interannual timescales, ENSO, AO, and SH, among others, have been shown to influence some of the variability in precipitation and circulation in Southern Asia (Gong et al. 2001; Gong and Ho 2002; Wu and Wang 2002; Yadav et al. 2009; Cannon et al. 2015). The main goal of this section is to explore how these different climate modes modulate the frequency and intensity of HMA ARs.

ENSO warm phases have been shown to increase precipitation in western Himalaya during the winter, attributed to increased convergence over southeastern Asia due to increased subsidence over the maritime continent (Yadav et al. 2009; Cannon et al. 2015). During the warm phase of ENSO, when the tropical oceans are warmer than usual, anomalous upper-level convergence over southern Asia has been shown to intensify WWDs (Yadav et al. 2009; Thapa et al. 2018). Rana et al. (2019) explains that increased precipitation in Central Southwest Asia during winter season El Niño years is mainly attributed to a deepened trough over Central Southwest Asia with cyclonic circulation that enhances southwesterly flow and moist air advection from the Indian Ocean basin into Central Southwest Asia. Guan and Waliser (2015) found that El Niño conditions slightly increase NDJFM AR frequency in southwest and southern Asia but has no considerable impact on precipitation. AR frequency was shown to decrease significantly in southwest Asia during La Niña years in NDJFM (Guan and Waliser 2015).

Here we evaluated the importance of the climate modes by calculating the difference in the proportion of HMA ARs for El Niño phase compared to La Niña phase, AO$+$ conditions compared to AO− conditions, SH$+$ conditions compared to SH− conditions and MJO conditions compared to no MJO conditions (see Table 1). For example, we compute the seasonal proportions of HMA ARs for El Niño and La Niña conditions during 1979–2019; the null hypothesis is that El Niño and La Niña frequencies are equal. This is evaluated using a z test at the 5% significance level (see Appendix C for equations). Significant differences at a 5% and 1% significance level in Table 1 are noted and indicate that Western and Northwestern HMA ARs are more frequent during El Niño compared to neutral and less frequent during La Niña conditions compared to neutral. However, ENSO related changes in frequency are considered significantly larger during La Niña conditions than during El Niño conditions. To investigate the impact of ENSO on the magnitude of IVT within the AR, we examined the distribution of the average IVT anomalies within the AR during the different types of HMA ARs for La Niña, neutral, and El Niño conditions (Fig. S8a). There does not appear to be any dramatic difference in the average IVT magnitude within the ARs when comparing El Niño, neutral, and La Niña conditions. Eastern HMA ARs during La Niña seem to have larger variability in AR IVT, but few outliers, whereas Eastern HMA ARs during El Niño have less variability but larger outliers.

**Table 1:** Z scores used to test the difference in the proportion of AR days during DJFMAM 1979–2019 between the conditions of various climate indices and neutral conditions

Conditions	Western HMA AR	Northwestern HMA AR	Eastern HMA AR	All AR days
El Niño vs. La Niña	2.74**	3.97**	0.96	5.07**
El Niño vs. Neutral	0.93	0.07	− 0.78	0.03
La Niña vs. Neutral	− 2.29*	− 4.46**	− 1.89	− 5.89**
AO$+$ vs. AO−	2.45*	5.24**	− 0.77	4.52**
AO$+$ vs. Neutral	3.18**	3.28**	− 2.91**	1.99*
AO− vs. Neutral	0.69	− 2.02*	− 2.1*	− 2.54*
SH$+$ vs. Neutral	− 0.05	0.02	− 2.02*	− 1.65
SH$+$ vs. SH−	− 1.98*	− 2.24*	0.05	− 2.76**
SH− vs. Neutral	1.79	2.11*	− 2.1*	0.91
MJO vs No MJO	1.35	− 1.54	− 0.7	− 0.71

*Statistically significant at the 5% significance level

**Statistically significant at the 1% significance level

**Fig. 9:**
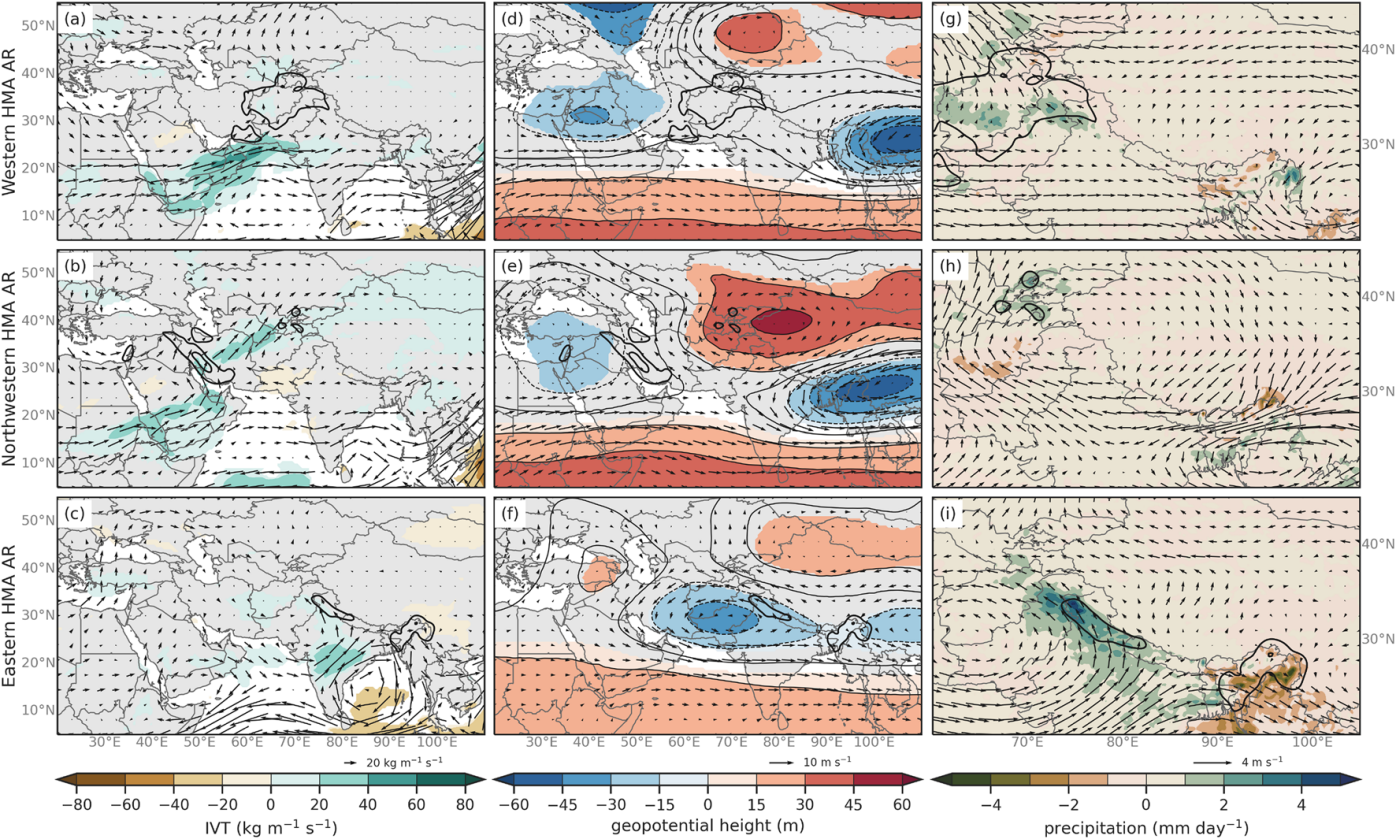
**a** Composite differences of IVT (shaded and vectors, kg m$^{-1}$ s$^{-1}$) for Western HMA AR (Type 1) El Niño and La Niña conditions. Only differences in IVT that are considered at or above the 95% confidence level are shaded. **b** Same as **a** but for Northwestern HMA ARs (Type 2). **c** Same as **a** but for Eastern HMA ARs (Type 3). **d** Composite differences of 250 hPa geopotential heights (shaded and contours, m) and winds (vectors, m s$^{-1}$) between HMA AR days for Western HMA AR (Type 1) El Niño and La Niña conditions based on ERA5 for 1979–2019. Only differences in heights that are considered at or above the 95% confidence level are shaded. **e** Same as **d** but for Northwestern HMA ARs (Type 2). **f** Same as **d** but for Eastern HMA ARs (Type 3). **g** Composite differences of precipitation (shaded, mm day$^{-1}$) and 500 hPa wind direction (vectors, m s$^{-1}$) for Western HMA ARs (Type 1) El Niño and La Niña conditions. **h** Same as **g** but for Northwestern HMA ARs (Type 2). **i** Same as **g** but for Eastern HMA ARs (Type 3). The thick black contours in all plots are showing the mean anomaly composite rainfall (mm day$^{-1}$) for their respective AR Type with intervals at 2 mm day$^{-1}$, 6 mm day$^{-1}$, and 10 mm day$^{-1}$

To understand why El Niño conditions favor increased frequency of Karakoram and Pamir ARs in contrast with La Niña, we compare DJFMAM averages of moisture and circulation fields for the two phases of the ENSO. Figures 9a, d, g shows the difference (El Niño minus La Niña) in the anomalies of IVT, 250 hPa geopotential height and winds, and precipitation and 500 hPa winds for Western HMA ARs and Fig. 9b, e, and h show differences for Northwestern HMA ARs. It is important to note that anomalies are relative to their respective AR Climatologies (see Fig. 8) and not the annual climatology. At upper levels, Western and Northwestern HMA ARs are related to weaker upper-level trough centered at 30$^{\circ }$ N and 40$^{\circ }$ E and a stronger ridge near 80$^{\circ }$ E during El Niño conditions compared to La Niña. This suggests that the El Niño response in Central Asia favors an anticyclonic flow centered North of the Hindu Kush for Western HMA ARs and over the Hindu Kush for Northwestern HMA ARs. This increases transport of moisture to the high latitudes during Western and Northwestern HMA ARs, increasing precipitation in the Karakoram and Hindu Kush during Western HMA ARs and increasing precipitation in the Pamir region during Northwestern HMA ARs. While the upper-level blocking during El Niño is an important difference, the increased IVT in the higher latitudes during El Niño compared to La Niña indicates that the increased Northwestern HMA AR frequency seems influenced by atmospheric conditions that favor the enhancement in water vapor transport to relatively higher latitudes.

The positive (negative) phase of AO is characterized by negative (positive) 1000 hPa geopotential height anomalies in the Arctic and two zonal bands of positive (negative) 1000 hPa geopotential height anomalies in the midlatitudes, over the Pacific and Atlantic Oceans (Thompson and Wallace 1998). Positive AO conditions encourage stronger upper-level westerlies, enhanced polar circulation, and the jet stream is typically located further poleward. Cannon et al. (2015) showed that in HMA, positive AO conditions are associated with a poleward shift of the eastern extent of the subtropical jet, and an increase in magnitude in the jet to the west of the Karakoram. They indicated a strong positive relationship between the positive phase of AO and increased 200 hPa zonal winds in the midlatitudes. These increased zonal winds modify the jet position and increase WWD activity and precipitation over HMA (Cannon et al. 2015). Increased precipitation in northwest India during the positive phase of AO has also been noted by other studies (Yadav et al. 2009; Thapa et al. 2018). The negative phase of AO is usually associated with a weaker polar vortex and weaker upper-level westerlies that allows the jet stream to undulate more, increasing cold air advection from the Arctic and has been attributed to increased storminess in the midlatitudes (Cannon et al. 2015).

**Fig. 10:**
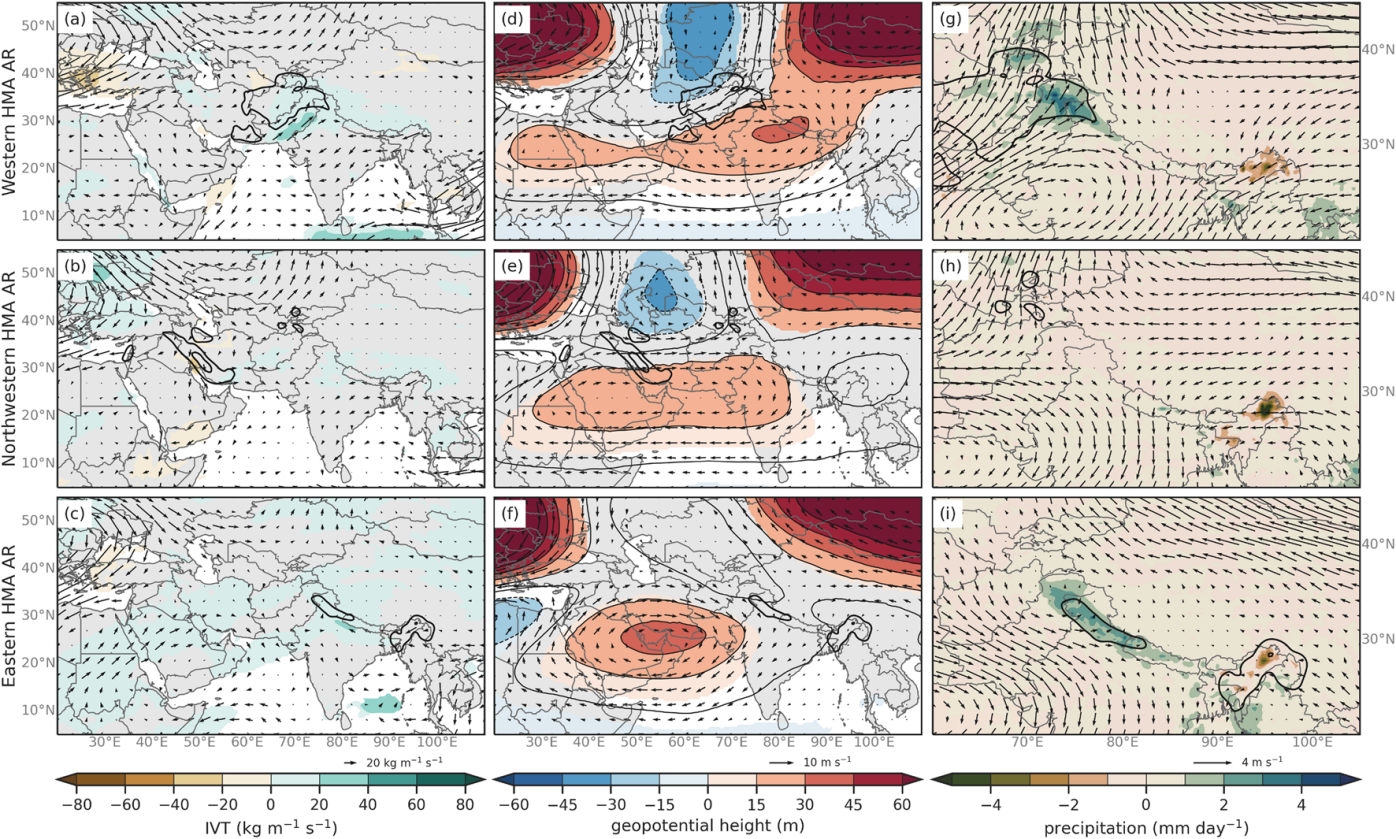
Same as Fig. 9, but for AO$+$ and AO− conditions

Z scores for differences in proportion of frequency of HMA ARs during AO$+$ and AO− conditions compared to neutral conditions indicate that Western and Nortwestern HMA ARs are significantly more frequent during positive AO conditions and less frequent during negative AO conditions compared to neutral. This is consistent with Guan and Waliser (2015), which found increased AR frequency over southwest Asia during the positive phase of AO, where Western and Northwestern HMA ARs typically enhance precipitation. Eastern HMA ARs are significantly less frequent during both AO$+$ and AO− conditions. Difference composites of 250 hPa geopotential height and winds, IVT, precipitation, and 500 hPa winds are shown in Fig. 10 for AO$+$ and AO−. All three AR types show increases in water vapor content between 20–40 kg m$^{-1}$ s$^{-1}$ along southwest Asia that could be attributed to an anticyclonic anomaly over the Arabian Sea (Fig. 10a–f). This may increase the amount of precipitation in the Karakoram and western Himalayas for Western and Eastern HMA ARs during AO$+$ conditions compared to AO− (see Fig. 10g, i). However, the anomalous trough and associated cyclonic circulation near the Tien Shan region during Western ARs implies higher than average wind speeds potentially increase precipitation in the Tien Shan during AO$+$ compared to AO−conditions (see Fig. 10g). The location of the ridges and troughs during AO$+$ compared to AO− and the lower difference in IVT suggests that dynamics (likely through enhanced orographic lifting caused by strong winds) play a more important role than thermodynamics (increase in water vapor content) in influencing AR frequency during AO conditions.

The Siberian High is a high-pressure center located in the northeastern part of Eurasia that has been shown to strongly influence processes from surface temperatures to the middle troposphere and thus the climate in middle to high Eurasia during winter (Gong et al. 2001; Gong and Ho 2002; Wu and Wang 2002). Weakening of the SH encourages disturbances in the weaker subtropical jet, allowing for WWD to propagate toward HMA, increasing precipitation in Central Himalayas (Cohen and Entekhabi 1999; Wu and Wang 2002; Cannon et al. 2015). Rana et al. (2019) shows that ridging that extends into central Asia and Europe during SH$+$ blocks eastward propagating westerly storm system, and restricts westerly flow to more southerly latitudes, resulting in increased precipitation in the southern region of Central Southwest Asia. Gong and Ho (2002) found that the SH explains 24% of the interannual variance in surface temperature over Eurasia, while AO explains roughly 30% of the variance, and that there are strong connections between AO and SH. They also found that 26% of precipitation variance over Eurasia was explained by AO, SH, and a few other prominent Eurasian climate modes, 9.8% of which SH contributed (Gong and Ho 2002). They conclude that after AO, the Siberian High is the second most important influence on temperature and precipitation in Eurasia, making it important to understand the influence of SH on HMA AR frequency.

**Fig. 11:**
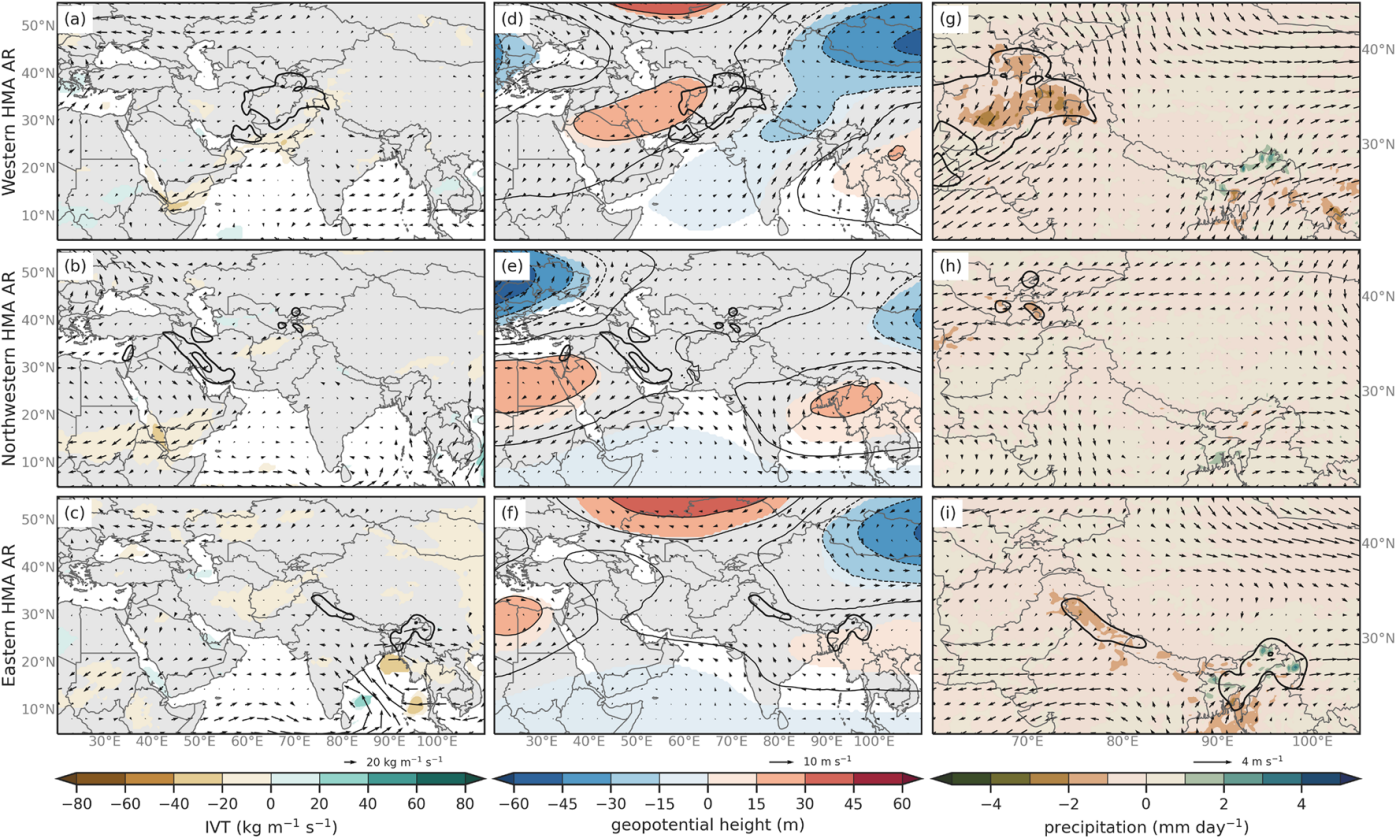
Same as Fig. 9, but for SH$+$ and SH− conditions

Table 1 indicates that Western and Northwestern HMA ARs are significantly more frequent during SH−compared to neutral. Figure 11a shows a significant decrease in water vapor content along the western basin of the Arabian Sea that is associated with enhanced ridging over Saudi Arabia during SH$+$ compared to SH negative during Western and Northwestern HMA ARs (Fig. 11b, e). Additionally, there is an increase in the magnitude of northwesterly winds around the Western Himalayas that would potentially decrease precipitation in these regions in SH$+$ compared to SH− (see Fig. 11g). Eastern HMA ARs are more frequent during SH$+$ compared to neutral (see Table 1), which is attributed to an anticyclonic anomaly in IVT over the Bay of Bengal increasing precipitation in Eastern Himalayas during SH$+$ conditions (see Fig. 11c, i).

The MJO, which is the primary mode of intraseasonal variability in the tropics (Madden and Julian 1971, 1972), has been shown to influence precipitation and circulation regimes in HMA as well as ARs across the globe by forcing a Rossby wave response due to changes in convection and diabatic heating (Barlow et al. 2005; Ralph et al. 2011; Guan et al. 2012; Guan and Waliser 2015; Cannon et al. 2017). Cannon et al. (2017) found that the MJO modifies the circulation and moisture availability within WWD, but no one phase favors extreme precipitation in High Mountain Asia. Guan and Waliser (2015) found that AR frequency increased in southwest Asia during NDJFM for MJO phases 1, 2, and 8 and only increased precipitation in HMA only during phase 8. They also found that AR frequency in southern Asia decreased significantly during MJO phases 4 and 5 with decreased precipitation in HMA during phase 5. According to z-scores from differences in proportion of HMA ARs, Eastern and Northwestern HMA ARs are less frequent when there is any active MJO phase compared to when there is no active MJO phase and Western HMA ARs are more frequent during any active MJO phase, but no significant differences were found (see Table 1). Examining each MJO phase as well as the differences in circulation and water vapor did not yield any significant results. Although previous studies with distinct methodologies (Barlow et al. 2005; Cannon et al. 2017) have suggested links between the MJO and extreme precipitation in HMA, our results did not point to any strong connection in HMA AR frequency and MJO phase. Understanding the complexities of the impact of MJO on ARs is beyond the scope of this current analysis, and future work is needed to make significant conclusions on MJO and HMA ARs.

## Discussion and conclusions

The objective of this research was to identify the climatology of ARs that influence HMA and investigate their linkages with large-scale circulation and precipitation patterns in the winter and spring seasons. Using ERA5 reanalysis and a global AR catalog, this study revealed the climatology of ARs that reach HMA, and their relationship with precipitation regimes in southern Asia using combined EOF analysis with k-means clustering. While there is on average little to no vapor flux in Southern Asia during the winter and spring seasons, enhanced IVT during HMA ARs results in roughly 40–60% of the total seasonal precipitation. Three AR subtypes penetrate far inland across Southern Asia, reach HMA during the winter and spring, and result in different precipitation patterns.

The three AR types are delineated by the location of their above-average precipitation. Western HMA ARs are associated with low geopotential heights directly north of the Arabian Sea that results in enhanced IVT and increased precipitation in Western Himalayas and the Karakoram. IVT composites for Western HMA ARs identify an anticyclonic circulation center over the Arabian Sea that is responsible for transporting water vapor poleward. Northwestern HMA ARs are characterized by a tilted trough axis and increased IVT that reaches as far as 40$^{\circ }$ N that increases precipitation in the Hindu Kush and the Pamirs. IVT during Northwestern HMA ARs has an anticyclonic circulation that covers most of the Arabian Sea and India. Eastern HMA ARs are associated with southwesterly moisture flux across the Bay of Bengal that results in above-average precipitation in Eastern Himalaya. Specifically, Eastern HMA ARs are associated with upper-level cyclonic circulation over the Tibetan Plateau and source moisture from the Arabian Sea and the Bay of Bengal regions related to anti-cyclonic circulation. Northwestern HMA ARs transition into Western HMA ARs 24% of the time, and Western HMA ARs transition into Eastern HMA ARs 18% of the time, indicating that while each subtype has its own unique characteristics, they are not all completely independent. Additionally roughly 15% of the time for any given AR type, there is a second AR located in the Southern Asia region. For example, if there is a Northwestern or Western HMA AR, there can also be an Eastern HMA AR 15% of the time, explaining the precipitation signatures in Eastern and Western HMA for all three synoptic composites.

Between 1979 and 2017, all three AR types have a history of being associated with extreme rainfall that can trigger flooding and/or landslide events that severely impacted the region. For example, in April 2016, an extreme rainfall event associated with a Northwestern HMA AR led to the occurrence of heavy flooding, lightning, and landslides in Kohistan, Pakistan, which resulted in over 100 fatalities (Kirschbaum et al. 2010). At least 56 landslides in the Global Landslide Catalog were associated with all three types of ARs (Kirschbaum et al. 2010). However, more landslides and extreme rainfall events may have been associated with HMA ARs, and a more thorough fine-scale analysis is needed to understand the meteorological influence of ARs on these extreme events.

This research further examined the relationship between HMA ARs and large-scale climate modes such as the ENSO, AO, and SH. The relationships between these modes and HMA ARs are complex, but we find evidence that Western and Northwestern HMA ARs are more frequent during El Niño, AO$+$, and SH− conditions. We also described the impacts of each climate mode on the broader circulation and water vapor patterns in this region. Increased frequency in Western and Northwestern HMA ARs during El Niño and AO$+$ is attributed to the location of the wave train, identified by signatures in the upper-level jet. During El Niño compared to La Niña, precipitation and IVT are more likely to be higher than average for both Western and Northwestern HMA ARs. During AO$+$ compared to AO−, precipitation is likely to increase in the Karakoram and Pamirs, but decrease in Eastern Himalayas. During SH$+$ compared to SH−, precipitation is likely to decrease in the Hindu Kush and Pamirs regions while increasing in the Eastern Himalayan regions. During SH$+$, an upper-level ridge over Southwest Asia can block eastward propagating westerlies, which can funnel upper-level winds to a more equatorward track and encourages precipitation to the east of the Karakoram. Relationships between climate modes further complicate these results and the understanding of their influence on circulation patterns. For example, during the winter and spring months in the 40 years of the study, there were only 25 days where an El Niño occurred without the influence of a different climate mode (e.g. AO or SH). While it is very clear that different climate modes influence the frequency of all three types of ARs, future work should consider exploring the influence of ENSO, AO, and the SH on HMA ARs.

This work quantified the contribution of HMA ARs to winter and spring precipitation and illustrates the importance of ARs to the hydrological cycle in HMA by determining the regional climatology of ARs in HMA, as well as exploring the dynamical processes that relate ARs to precipitation regimes in Southern Asia. In addition to our findings regarding conditions of three different ARs and their interactions with the HMA, our research points to a broader consideration: the importance of studying ARs in novel sites where ARs penetrate inland and can contribute greatly to the regional hydroclimate. The characterization of the HMA AR subtypes can be applied to future work to examine the mesoscale and thermodynamic characteristics of HMA ARs and exploring the long-term changes in the large-scale dynamics related to ARs. This can be used to uncover the relationship between ARs and local hazards such as landslides, floods, and other influences on HMA climate. Future modeling work to improve forecasting skill for ARs in this region could benefit from knowing the synoptic characteristics associated with each type of HMA AR and their potential impacts on regional precipitation. Understanding the role of ARs in local hydrology and the large-scale dynamics driving the ARs inland is critical to minimize uncertainty regarding the future of water resources in Southern Asia.

### Supplementary Information

Below is the link to the electronic supplementary material.

Supplementary file1 (pdf 11214 KB)

## Data Availability

The AR data were provided by Bin Guan via https://ucla.box.com/ARcatalog. Development of the AR detection algorithm and databases was supported by NASA. ERA5 data on single levels (Hersbach et al. 2018b, https://cds.climate.copernicus.eu/cdsapp#!/dataset/10.24381/cds.adbb2d47) and pressure levels (Hersbach et al. 2018a, https://cds.climate.copernicus.eu/cdsapp#!/dataset/10.24381/cds.bd0915c6) were downloaded from the Copernicus Climate Change Service (C3S) Climate Data Store. The results contain modified Copernicus Climate Change Service information 2020. Neither the European Commission nor ECMWF is responsible for any use that may be made of the Copernicus information or data it contains. MERRA2 data (Global Modeling and Assimilation Office (GMAO) 2015; Gelaro et al. 2017, https://disc.gsfc.nasa.gov/datasets/M2I6NPANA_V5.12.4/summary?keywords=MERRA2), IMERG-PM v06 data (Huffman et al. 2019, https://disc.gsfc.nasa.gov/datasets/GPM_3IMERGDF_06/summary), APRHODITE data (Yatagai et al. 2012; Maeda et al. 2020, http://aphrodite.st.hirosaki-u.ac.jp/products.html) and NOAA topography data (Amante and Eakins 2009, https://www.ncei.noaa.gov/access/metadata/landing-page/bin/iso?id=gov.noaa.ngdc.mgg.dem:316) are all freely available online. The ENSO and AO indices were calculated by the National Weather Service Climate Prediction Center and can be found at https://origin.cpc.ncep.noaa.gov/products/analysis_monitoring/ensostuff/ONI_v5.php and https://www.cpc.ncep.noaa.gov/products/precip/CWlink/daily_ao_index/monthly.ao.index.b50.current.ascii.table. The SH index was calculated using ERA5 mean sea level pressure data and the methods outlined in Panagiotopoulos et al. (2005). The MJO-RMM index can be found at (Wheeler and Hendon 2004, http://www.bom.gov.au/climate/mjo/graphics/rmm.74toRealtime.txt). The MJO index referenced in Jones (2009) was provided by Charles Jones. The Westerly Disturbance Catalog from Hunt et al. (2018) can be found in the CEDA archive at (Turner and Hunt 2019, http://catalogue.ceda.ac.uk/uuid/b1f266c25cf2445f8b87d874f6ac830a). The Global Landslide Catalog from (Kirschbaum et al. 2010, 2015) can be found at https://data.nasa.gov/Earth-Science/Global-Landslide-Catalog-Export/dd9e-wu2v.

## Appendix A: cEOF Analysis

For each of the ARs within DJFMAM, ERA5 meridional and zonal integrated water vapor transport (IVT) were subset to 20$^{\circ }$ E to 100$^{\circ }$ E and 10$^{\circ }$ N to 50$^{\circ }$ N. Next, the mean annual cycle (computed using the first two harmonics) of the atmospheric fields were subtracted, each grid cell was weighted by the square root of the cosine of the latitude, and the long-term mean was removed from the data. Variables were normalized by the standard deviation in space and time. This standardization transformed the 3-dimensional data into non-dimensional data with a standard deviation of one, which is necessary in cEOF for variables with different units. The data were then reshaped into a rectangular matrix Z, with dimensions of 3NM x n, where N and M are the numbers of grid points in the X and Y directions, m is the total number of grid points (i.e. $m =NxM$), and n is the number of separate AR cases. The similarity matrix (R) of Z was computed using the formula:

1 $$\begin{aligned} R = \frac{1}{NM - 1} Z_T Z \end{aligned}$$

where R is the similarity matrix, N and M are the numbers of grid points in the X and Y directions, and Z is the nondimensional data with a standard deviation of 1. The similarity matrix for the spatial correlations between atmospheric fields was calculated from different snapshots in time (t-mode EOF), resulting in an *mxm* similarity matrix rather than temporal correlations between grid points (s-mode EOF), which results in an *nxn* similarity matrix (Compagnucci and Richman 2008). The choice to use t-mode EOF versus s-mode EOF (spatial versus temporal correlations) was made to get objective comparisons between AR cases and differentiate between the different synoptic types of ARs in HMA. For more discussion on t-mode versus s-mode EOF, see Compagnucci and Richman (2008); Mercer et al. (2012). Eigenvector decomposition (V) of the similarity matrix, R, was calculated giving the time coefficients for the data. Spatial loadings (cEOFs) were extracted by matrix multiplying the standardized, non-dimensional data (Z) by the eigenvectors (V).

## Appendix B: K-means cluster analysis

K-means clustering is a form of iterative, unsupervised learning that classifies the data without having been previously trained (Wilks 2019b). First, the number of clusters (k) is specified by the user and the algorithm randomly places cluster centers or centroids among the data points. Then each data point is assigned to the closest cluster by calculating its Euclidean distance with respect to each centroid. Once each data point is assigned to a cluster, then new centroids are calculated by computing the average of the data points assigned to that cluster. The data points are then reassigned to clusters based on the new centroid locations, and new centroid locations are determined. This process repeats until the data points are no longer assigned to a different cluster. The number of clusters is a subjective choice, so for this analysis, we tested k = 2 through k = 15 and chose the level of clustering that maximized similarity within clusters and minimized similarity between clusters. To determine the number of clusters that maximized similarity within clusters, we visually inspected the kernel density function of the first two cEOFs to identify potential maxima prior to k-means analysis. Silhouette scores were reiteratively calculated for clusters k = 2 through k = 15 to determine the optimal number of clusters that minimized similarity between clusters. The mean silhouette score describes how close each point in one cluster is to points in the other clusters, with a score of 1 indicating that the points in the cluster are far away from the other clusters (Wilks 2019b). Last, we also visually inspected the resulting cluster synoptic composite maps to confirm that the choice in the number of clusters minimized similarity between clusters and maximized similarity within clusters.

## Appendix C: Z-score tests

To test the differences in the proportion of the different types of ARs between the positive, negative, and neutral conditions of climate modes, we test the difference in two population proportions using z-scores (Spiegel 2013). For example, we test the differences in the proportion of the frequency of Eastern HMA ARs that are El Niño and the frequency of Eastern HMA ARs that are La Niña). The null hypothesis is that the proportion of sample 1 (Eastern HMA ARs during El Niño) is equal to the proportion of sample 2 (Eastern HMA ARs during La Niña).

2 $$\begin{aligned} Z = \frac{({\hat{p}}_{1} - {\hat{p}}_{2}) - 0}{\sqrt{({\hat{p}}(1-{\hat{p}})(\frac{1}{n_{1}} + \frac{1}{n_{2}})}} \end{aligned}$$

and

3 $$\begin{aligned} {\hat{p}} = \frac{Y_{1} + Y_{2}}{n_{1} + n_{2}} \end{aligned}$$

where $n_1$ and $n_2$ are the total number of observations in each sample (e.g., $n_1$ = total number of days that are considered El Niño during DJFMAM between 1979–2019), $Y_1$ is the number of the days considered Eastern HMA ARs and El Niño, $Y_2$ is the number of days considered Eastern HMA ARs and La Niña, ${\hat{p}}_{1}$ is the proportion of the sample 1 days to the number of days that are El Niño in DJFMAM between 1979–2019. ${\hat{p}}_{2}$ is the proportion of the sample 2 days to the number of days that are La Niña in DJFMAM between 1979–2019.

To test the difference of the means of the circulation and moisture variables, we used the z-score to test the null hypothesis that the sample means of sample 1 and sample 2 are equal (Spiegel 2013; Wilks 2019c). For example, sample 1 are the days in which there is an Eastern HMA AR with El Niño conditions, and sample 2 are the days in which there is an Eastern HMA AR with La Niña conditions. We used the z-score given by the following equations to test the null hypothesis at 95% significance level:

4 $$\begin{aligned} z = \frac{{\bar{X}}_{1} - {\bar{X}}_{2}}{\sigma _{{\bar{X1}} - {\bar{X2}}}} \end{aligned}$$

and

5 $$\begin{aligned} \sigma _{{\bar{X1}}- {\bar{X2}}} = \sqrt{\frac{\sigma ^{2}_{1}}{n_{1}} + \frac{\sigma ^{2}_{2}}{n_{2}}} \end{aligned}$$

where ${\bar{X}}_{1}$ and ${\bar{X}}_{2}$ are the sample means, $\sigma _{1}$ and $\sigma _{2}$ are the sample standard deviations, and $n_1$ and $n_2$ are the total number of observations in each sample.

## Declaration

### Code availability

The code for this analysis can be found at https://github.com/dlnash/AR_types.
