# Supplementary material for: Winter and spring atmospheric rivers in High Mountain Asia: climatology, dynamics, and variability
Source: Clim Dyn. 2021 Oct 24;58(9-10):2309–31. doi: 10.1007/s00382-021-06008-z (PMC9054897; doi:10.1007/s00382-021-06008-z)
Supplement: Supplementary file 1 — Supplementary file1 (pdf 11214 KB) [file 382_2021_6008_MOESM1_ESM.pdf]

Noname manuscript No.
(will be inserted by the editor)

Supporting Information for "Winter and Spring Atmospheric Rivers in High Mountain Asia: Climatology, Dynamics, and Variability"

Deanna Nash · Leila M.V. Carvalho · Charles Jones · Qinghua Ding

the date of receipt and acceptance should be inserted later

1 Supplemental Figures

D. Nash
Department of Geography
University of California
Santa Barbara, CA 93106
E-mail: dlnash@ucsb.edu

L.M.V Carvalho, C. Jones and Q. Ding
Department of Geography
and
Earth Research Institute
University of California
Santa Barbara, CA 93106

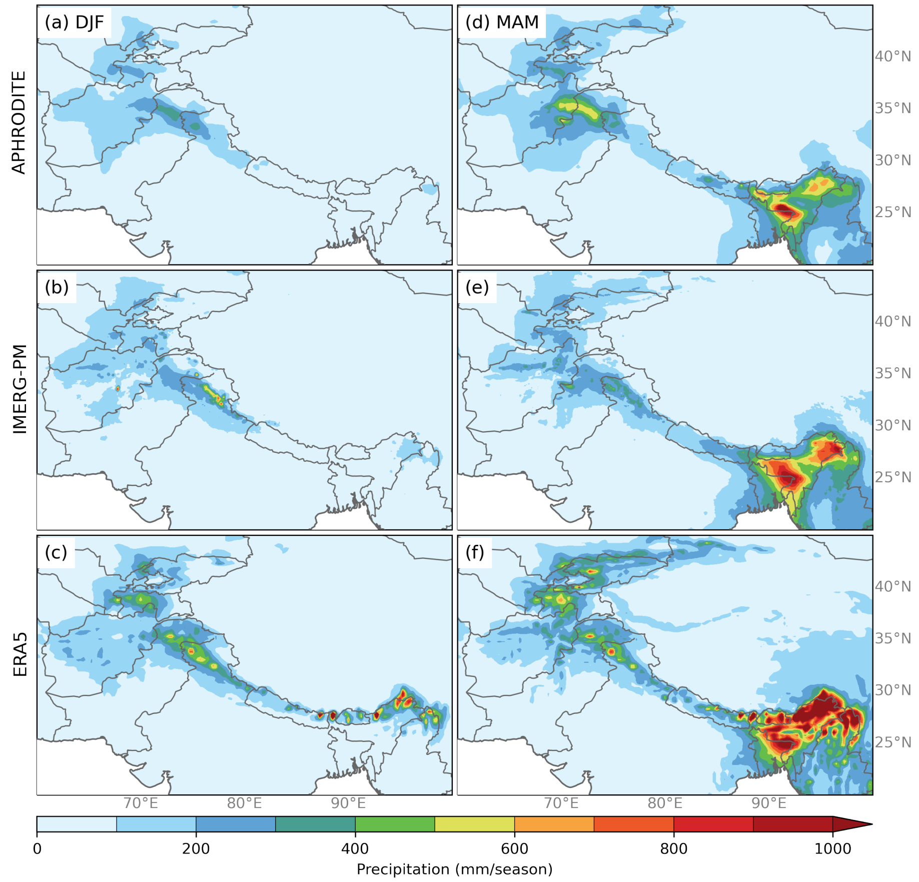

Fig. S1 The average seasonal (DJF - left and MAM - right) precipitation totals (shaded; mm season^{-1}) for APHRODITE (top row), IMERG-PM (middle row), and ERA5 (bottom row) data sources between December 2000 and March 2015.

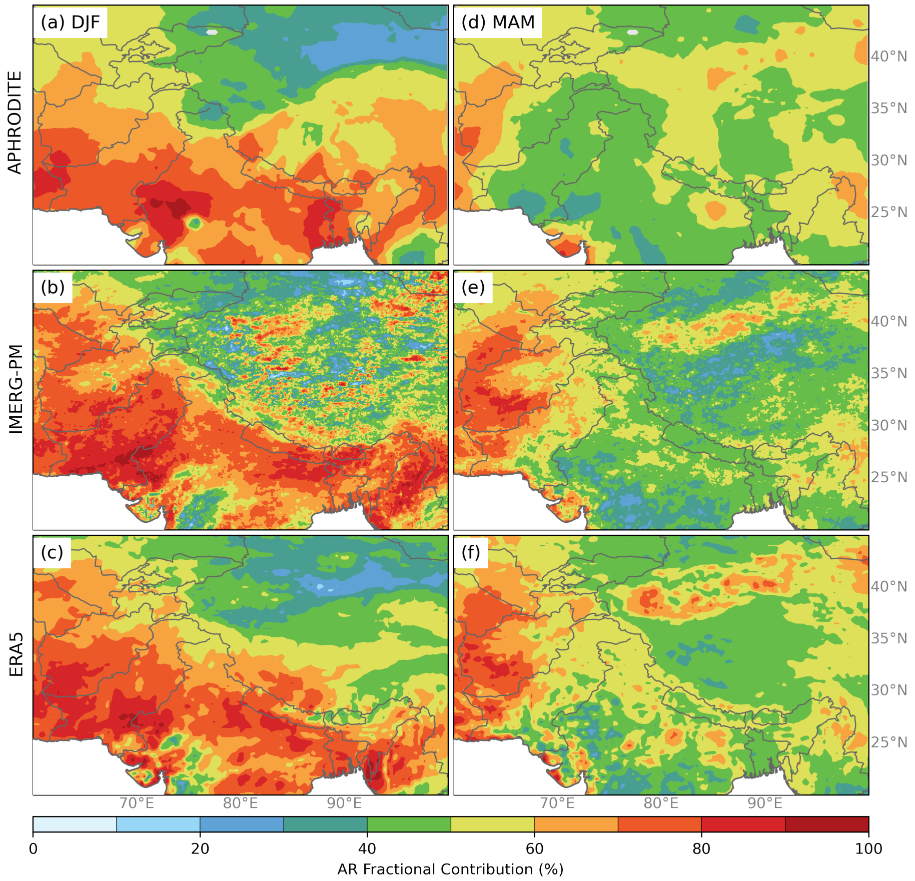

Fig. S2 The seasonal (DJF - left and MAM - right) AR precipitation fraction (shaded, %) for APHRODITE (top row), IMERG-PM (middle row), and ERA5 (bottom row) data sources between December 2000 and March 2015.

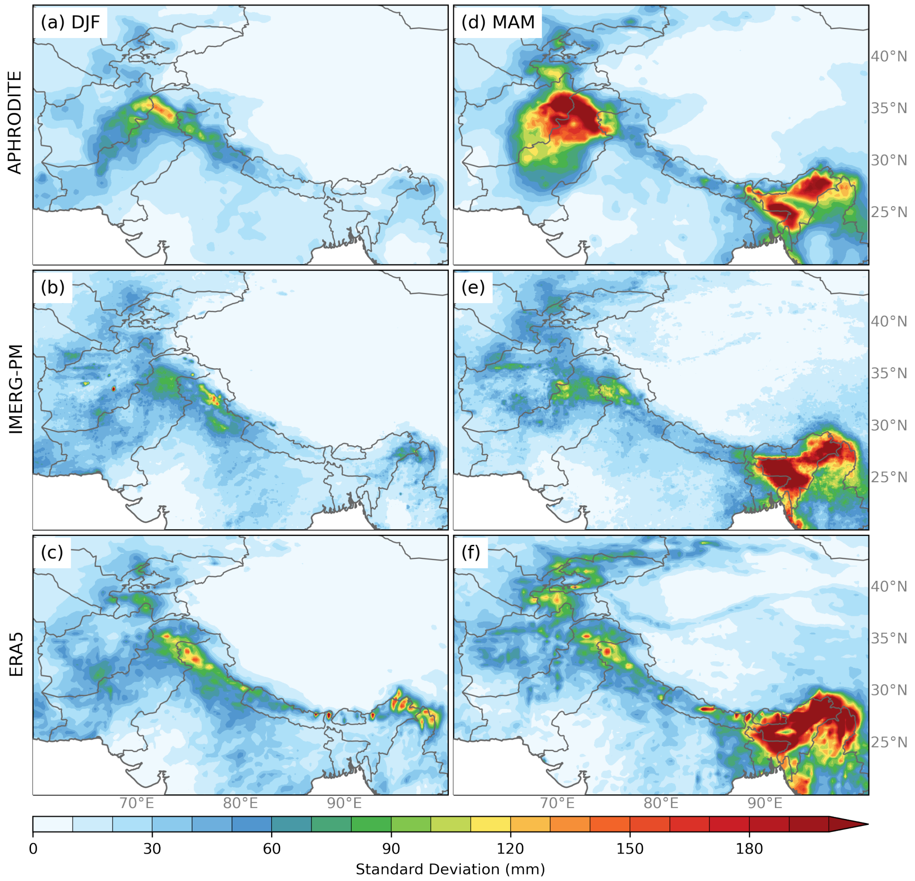

Fig. S3 The standard deviation (DJF - left and MAM - right) of precipitation during AR days (shaded; mm) for APHRODITE (top row), IMERG-PM (middle row), and ERA5 (bottom row) data sources between December 2000 and March 2015.

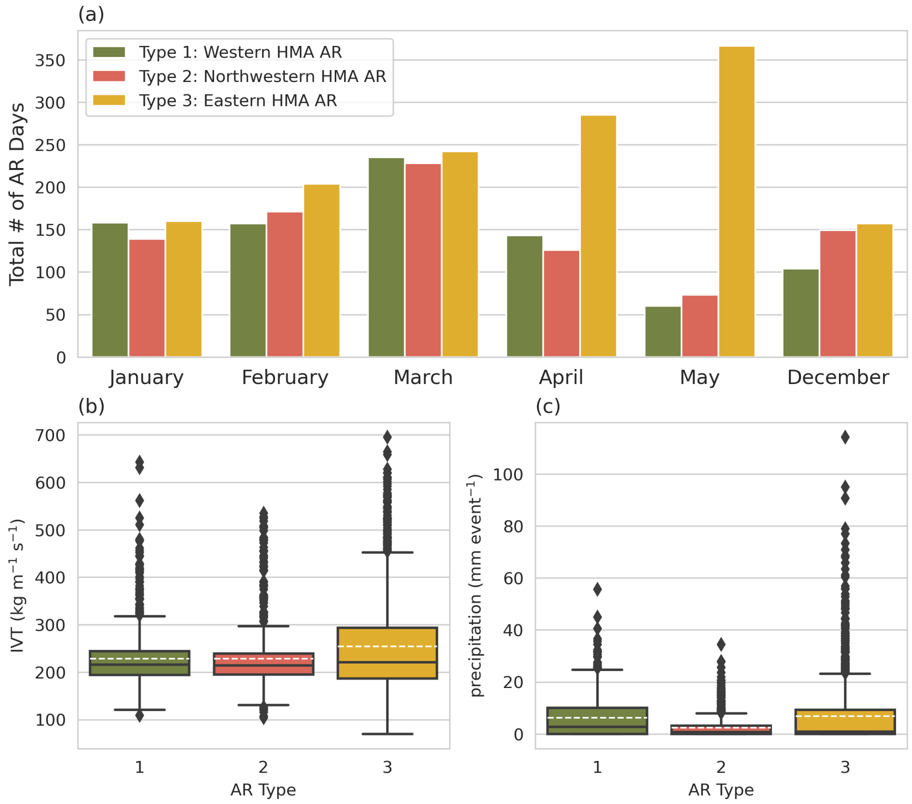

Fig. S4 (a) Frequency of the different AR Types during each month in DJFMAM. (b) Average IVT (kg m^{-1} s^{-1}) within ARs during Western HMA ARs (Type 1), Northwestern HMA ARs (Type 2), and Eastern HMA ARs (Type 3). The box extends from lower to upper quartiles of the data with a black line at the median and white dotted line at the mean. The whiskers show the range of the data and outliers are shown as points past the end of the whiskers. (c) The same as (b) but for weighted, area-averaged precipitation (mm event^{-1}) within a bounding box near where we see above-average precipitation for each AR Type (e.g. Type 1: Western HMA ARs (71-79°E, 32-37°N); Type 2: Northwestern HMA ARs (66-74°E, 37-40°N); Type 3: Eastern HMA ARs (90-100°E, 24-30°N).

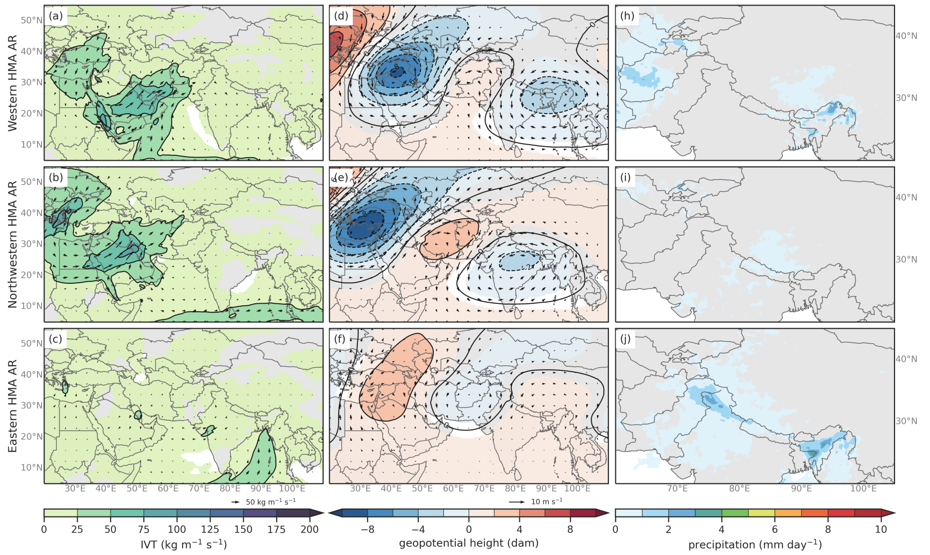

Fig. S5 Lead 2 DJFMAM average anomaly composites of (left column) IVT (shaded, contours, kg m^{-1} s^{-1}), (middle column) 250 hPa wind speeds (shaded and vectors; m s^{-1}) and 250 hPa geopotential height (contours; dam), and (right column) precipitation (shaded; mm day^{-1}) for Western HMA ARs (Type 1, first row), Northwestern HMA ARs (Type 2, second row), and Eastern HMA ARs (Type 3, third row). Only values that are considered statistically significant at the 95% confidence interval are shaded.

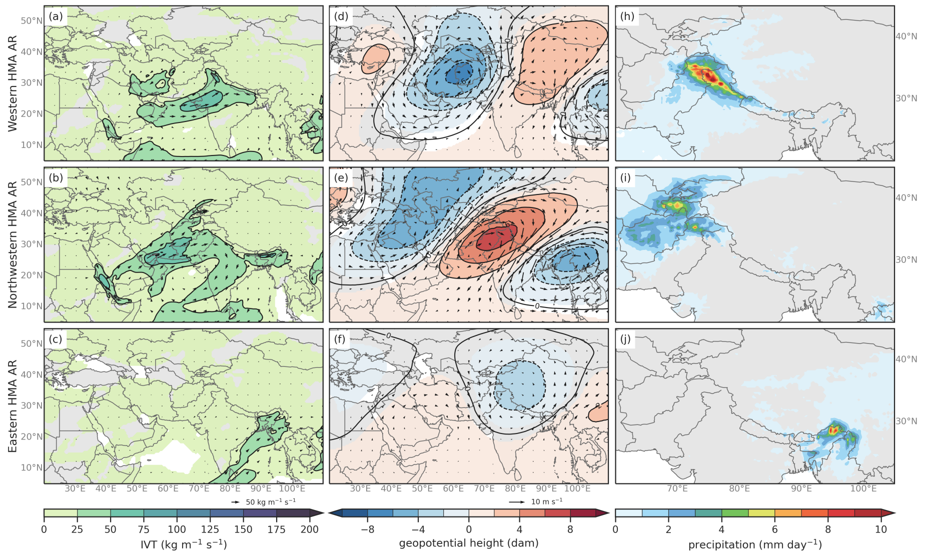

Fig. S6 Lag 2 DJFMAM average anomaly composites of (left column) IVT (shaded, contours, kg m^{-1} s^{-1}), (middle column) 250 hPa wind speeds (shaded and vectors; m s^{-1}) and 250 hPa geopotential height (contours; dam), and (right column) precipitation (shaded; mm day^{-1}) for Western HMA ARs (Type 1, first row), Northwestern HMA ARs (Type 2, second row), and Eastern HMA ARs (Type 3, third row). Only values that are considered statistically significant at the 95% confidence interval are shaded.

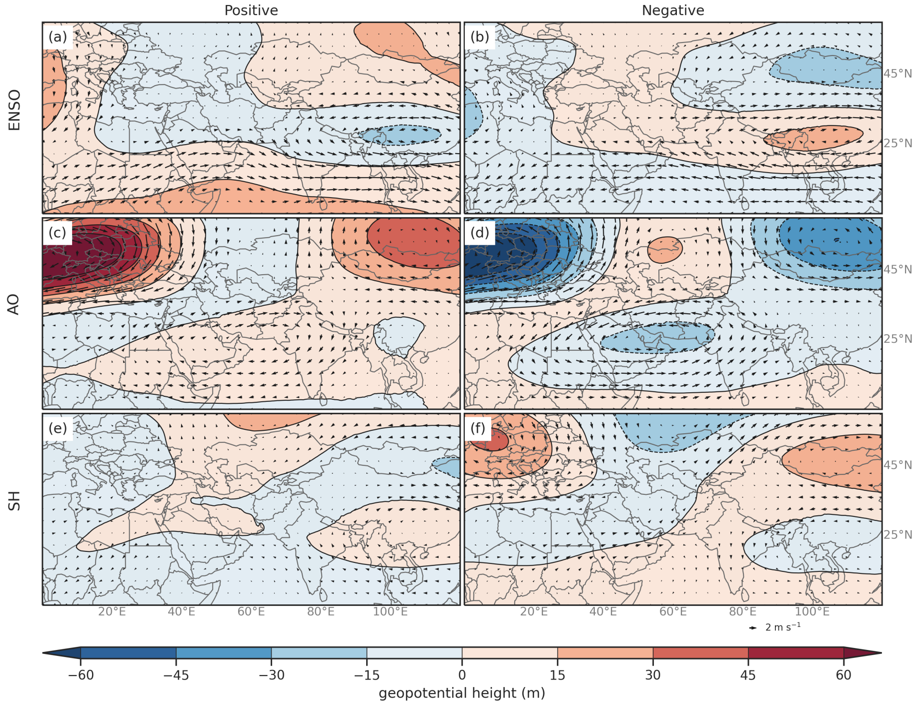

Fig. S7 Composites of 250 hPa geopotential height (shaded, contours, m) and winds (vectors, m s^{-1}) for all days in DJFMAM between 1979 and 2019 for (a) El Nino, (b) La Nina, (c) AO+, (d) AO-, (e) SH+, and (f) SH- conditions.

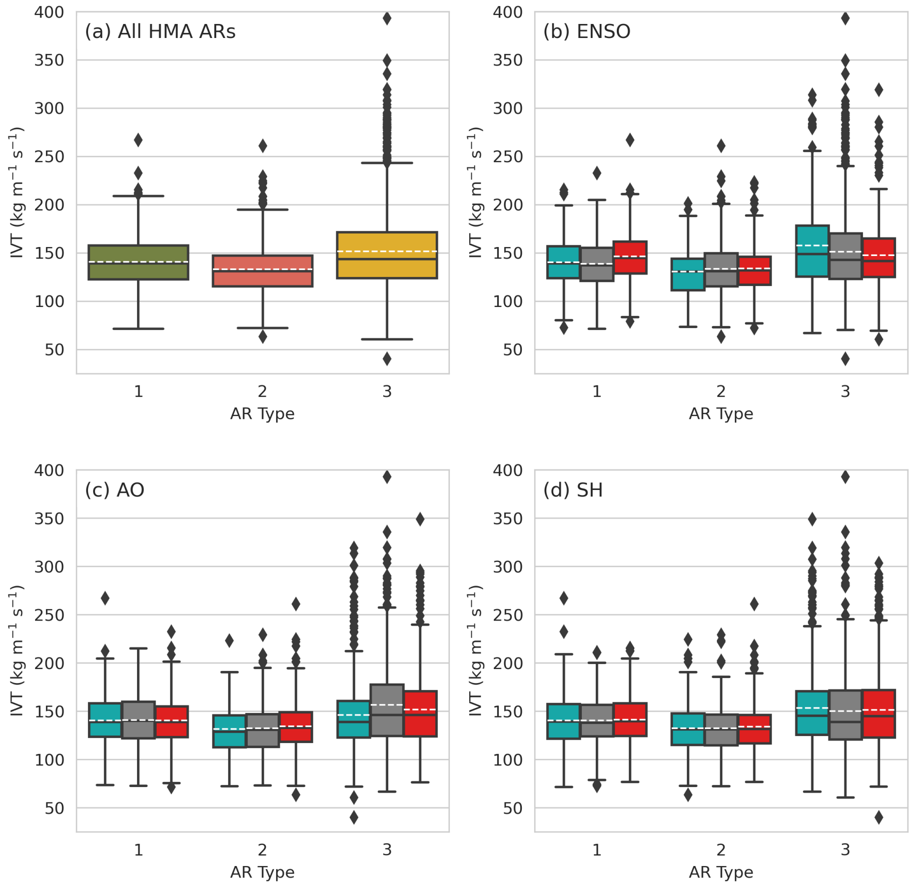

Fig. S8 (a) Average IVT anomalies within ARs during Western HMA ARs (Type 1), Northwestern HMA ARs (Type 2), and Eastern HMA ARs (Type 3). The box extends from lower to upper quartiles of the data with a black line at the median and white dotted line at the mean. The whiskers show the range of the data and outliers are shown as points past the end of the whiskers. (b) The same as (a) but broken down by ENSO negative (blue), neutral (grey), and positive (red) conditions. (c) The same as (b) but for AO. (d) The same as (b) but for SH.

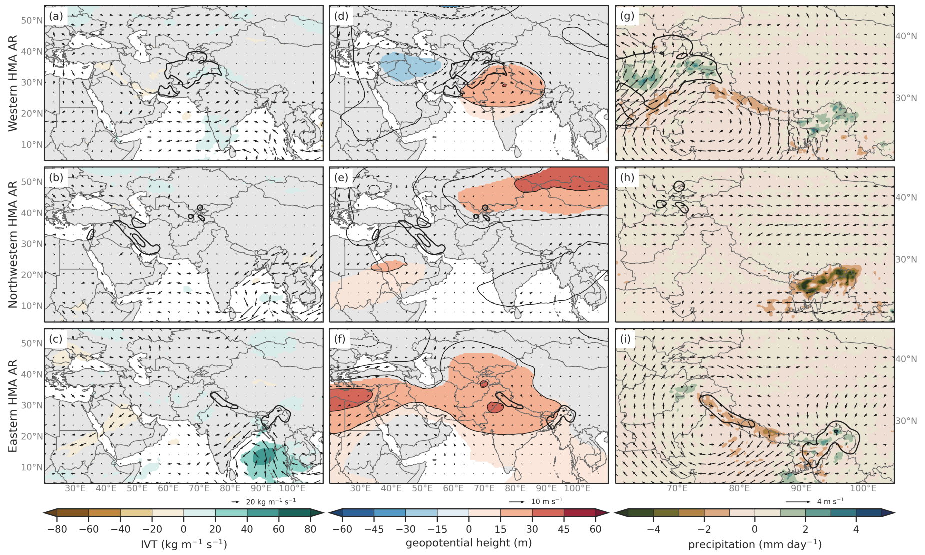

Fig. S9 (a) Composite differences of IVT (shaded and vectors, kg m^{-1} s^{-1}) for Western HMA ARs (Type 1) MJO and non-MJO conditions. Only differences in IVT that are considered at or above the 95% confidence level are shaded. (b) Same as (a) but for Northwestern ARs (Type 2). (c) Same as (a) but for Eastern HMA ARs (Type 3). (d) Composite differences of 250 hPa geopotential heights (shaded and contours, m) and winds (vectors, m s^{-1}) between HMA AR days for Western HMA ARs (Type 1) MJO and non-MJO conditions based on ERA5 for 1979-2019. Only differences in heights that are considered at or above the 95% confidence level are shaded. (e) Same as (d) but for Nortwestern HMA ARs (Type 2). (f) Same as (d) but for Eastern HMA ARs (Type 3). (g) Composite differences of precipitation (shaded, mm day^{-1}) and 500 hPa wind direction (vectors, m s^{-1}) for Western HMA ARs (Type 1) MJO and non-MJO conditions. (h) Same as (g) but for Northwestern HMA ARs (Type 2). (i) Same as (g) but for Eastern HMA ARs (Type 3). The thick black contours in all plots are showing the mean anomaly composite rainfall (mm day^{-1}) for their respective AR Type with intervals at 2 mm day^{-1}, 6 mm day^{-1}, and 10 mm day^{-1}.
